# Response of Methicillin-Resistant *Staphylococcus aureus* to Amicoumacin A

**DOI:** 10.1371/journal.pone.0034037

**Published:** 2012-03-30

**Authors:** Amrita Lama, Jan Pané-Farré, Tai Chon, Anna M. Wiersma, Clarissa S. Sit, John C. Vederas, Michael Hecker, Michiko M. Nakano

**Affiliations:** 1 Division of Environmental and Biomolecular Systems, Institute of Environmental Health, Oregon Health & Science University, Beaverton, Oregon, United States of America; 2 Institute for Microbiology, Ernst-Moritz-Arndt-University Greifswald, Greifswald, Germany; 3 Department of Chemistry, University of Alberta, Edmonton, Alberta, Canada; Charité-University Medicine Berlin, Germany

## Abstract

Amicoumacin A exhibits strong antimicrobial activity against methicillin-resistant *Staphylococcus aureus* (MRSA), hence we sought to uncover its mechanism of action. Genome-wide transcriptome analysis of *S. aureus* COL in response to amicoumacin A showed alteration in transcription of genes specifying several cellular processes including cell envelope turnover, cross-membrane transport, virulence, metabolism, and general stress response. The most highly induced gene was *lrgA*, encoding an antiholin-like product, which is induced in cells undergoing a collapse of Δψ. Consistent with the notion that LrgA modulates murein hydrolase activity, COL grown in the presence of amicoumacin A showed reduced autolysis, which was primarily caused by lower hydrolase activity. To gain further insight into the mechanism of action of amicoumacin A, a whole genome comparison of wild-type COL and amicoumacin A-resistant mutants isolated by a serial passage method was carried out. Single point mutations generating codon substitutions were uncovered in *ksgA* (encoding RNA dimethyltransferase), *fusA* (elongation factor G), *dnaG* (primase), *lacD* (tagatose 1,6-bisphosphate aldolase), and SACOL0611 (a putative glycosyl transferase). The codon substitutions in EF-G that cause amicoumacin A resistance and fusidic acid resistance reside in separate domains and do not bring about cross resistance. Taken together, these results suggest that amicoumacin A might cause perturbation of the cell membrane and lead to energy dissipation. Decreased rates of cellular metabolism including protein synthesis and DNA replication in resistant strains might allow cells to compensate for membrane dysfunction and thus increase cell survivability.

## Introduction

*Staphylococcus aureus* is the etiological agent for a large number of human infections, including pneumonia, meningitis, toxic shock syndrome, bacteremia, and endocarditis. *S. aureus* is notorious for developing rapid resistance to antibiotics, which is caused mainly by antibiotic selection and horizontal transfer of resistance genes [1]. Most notably, methicillin-resistant *S. aureus* (MRSA) has emerged quickly due to acquisition of the novel penicillin-binding protein 2A (PBP2A) encoded by *mecA* [2]. The *mecA* gene likely originated from *Staphylococcus sciuri* that is naturally sensitive to β-lactam [3] and *mecA* incorporated into a staphylococcal cassette chromosome (SCC*mec*) serves as a mobile genetic element, thus mediating transfer of the *mecA* gene into staphylococcal species [4]. Morbidity caused by MRSA infections per year in the U.S.A. was reported to be comparable to that caused by HIV/AIDS [5], which highlights the importance of this deadly pathogen. The glycopeptide vancomycin, the most commonly used antibiotic for the treatment of MRSA infections, is no longer effective enough because of the emergence of vancomycin-intermediate *S. aureus* (VISA) [6]. Therefore, there is an urgent need to find antibacterial agents with novel mechanisms of action and to understand how *S. aureus* acquires antibiotic resistance.

**Figure 1:**
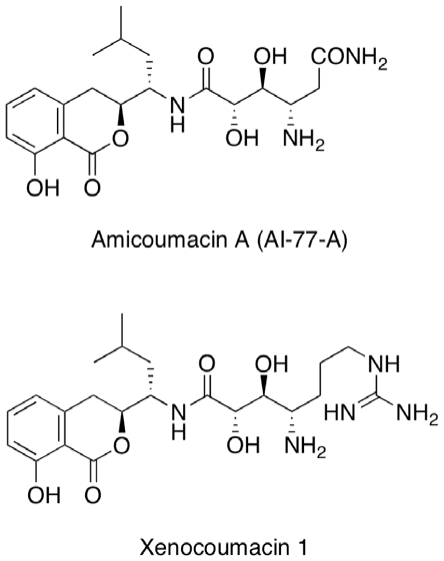
The chemical structures of amicoumacin A and xenocoumacin 1.

In this study, amicoumacin A, which possesses strong anti-MRSA activity, was investigated. Amicoumacin A, also known as AI-77-A, is one of numerous structurally related isocoumarin antibiotics [7], [8], [9], [10]. Other examples of this class of antibiotics include the xenocoumacins, the bacilosarcins, Sg17-1-4, PM-94128, and Y-05460 [11], [12], [13], [14], [15]. Figure 1 illustrates the structures of amicoumacin A and xenocoumacin 1. These isocoumarins exhibit a wide variety of biological effects, ranging from anti-bacterial to anti-tumor activity [11], [12], [13], [14], [15]. Amicoumacin A has anti-microbial, anti-inflammatory, and anti-ulcer properties [7], and anti-*Helicobacter* activity was later reported [16]. Despite the versatile activities, the mode of action remains unknown. Antibiotics currently used for MRSA infections either inhibit bacterial protein synthesis (linezolid) [17] or target the cell membrane (daptomycin) [18]. Amicoumacin A was reported to exhibit anti-MRSA activity [19]. This work has been aimed at elucidating the mechanism of action of amicoumacin A by examining genome-wide transcription induced by exposure of MRSA to amicoumacin A and identifying genetic changes that lead to reduced susceptibility to amicoumacin A.

## Results

### Isolation and identification of *Bacillus pumilus* strain C9 with anti-MRSA activity

A collection of 60 bacterial strains isolated from the Columbia River Estuary was screened for their potential antimicrobial activity against *S. aureus*. Three isolates exhibited clear inhibitory zones and were identified as *Pseudoalteromonas tunicata*, *Peudoalteromonas* sp., and *B. pumilus* based on the analysis of 16S rRNA gene sequence. Among the three strains, *B. pumilus* (named the strain C9) had the strongest activity against methicillin-sensitive *S. aureus* (MSSA) and MRSA and was chosen for further investigation.

### Purification and characterization of amicoumacin A

To identify its structure, the anti-MRSA substance was purified by methanol extraction, anion exchange column chromatography and RP-HPLC. The molecular mass of the compound was determined to be 424.2077 Da as determined by nanoESI FTICR mass spectrometry. FTICR analysis also revealed a plausible chemical formula (C_20_H_30_N_3_O_7_) for the substance. Through NMR analysis of the compound, chemical shifts and coupling constants were assigned for the protons in the molecule. Comparisons of the molecular mass, molecular formula and NMR data with the literature suggested that this substance is identical to amicoumacin A [7] (Figure 1).

Seven *B. pumilus* strains obtained from the *Bacillus* Genetic Stock Center were also checked for their anti-MRSA activity. No activity was detected from these strains except that 8A2 showed a weak anti-MRSA activity. However, the antibacterial spectrum of the compound from 8A2 significantly differed from that of the substance from C9 (data not shown). On the other hand, a similar anti-MRSA activity was detected in *B. pumilus* SAFR-032, a strain originally recovered from the Jet Propulsion Lab (Pasadena, CA) spacecraft assembly facility [20]. The results suggested that productivity of amicoumacin A was lost during domestication of *B. pumilus* strains and in fact, we have previously found a similar phenomenon for surfactin production in *B. subtilis* 168. In this case, introduction of *sfp* into domesticated *B. subtilis* strains restored surfactin production [21]. The *sfp* gene encodes 4′-phosphopantetheine transferase essential for nonribosomal peptides (NRP) and polyketides (PK) synthesis [22]. A recent study showed that PK and NRP synthesis genes are required for the production of xenocoumacin I [23], indicating that amicoumacin A is likely synthesized by a PK/NRP complex. It is conceivable that *B. pumilus* domesticated strains contain inactive *sfp* and hence are incapable of producing amicoumacin A. We designed primers using the *sfp* sequence of *B. pumilus* SAFR032 strain and used the primers to detect a PCR product with chromosomal DNA isolated from C9, 8A3, and 14A1. The PCR product with a high sequence homology to *sfp* from SAFR032 was detected in C9, but not in 8A3 and 14A1 (unpublished results), which is consistent with the notion that amicoumacin A is likely synthesized by a similar mechanism and the lack of an intact *sfp* is partially (if not solely) responsible for the loss of amicoumacin A production in domesticated *B. pumilus* strains.

### Antimicrobial spectrum of amicoumacin A

In order to evaluate the antimicrobial effect of amicoumacin A, we examined the spectrum of amicoumacin A. Two gram-negative bacteria, *E. coli* and *Pseudomonas aeruginosa* were resistant as were three gram-positive bacteria, *B. pumilus*, *Bacillus subtilis*, and *Listeria monocytogenes*. Among the susceptible gram-positive bacteria, *Bacillus anthracis*, *Bacillus cereus*, and *Bacillus thuringiensis* showed a moderate sensitivity. These bacteria exhibited a fairly large zone of inhibition (∼2 cm in diameter) after 4–5 h of incubation with amicoumacin A; however, several resistant colonies appeared after overnight incubation and the inhibition zone became smaller (0.5 cm in diameter). In contrast, amicoumacin A showed highly potent activity (inhibition zone >2 cm in diameter after overnight incubation) against all MSSA and MRSA strains tested including hospital-acquired MRSA (HA-MRSA), community-acquired MRSA (CA-MRSA), and Mu50, a HA-MRSA/VISA strain. In addition, *Staphylococcus carnosus* and *Streptococcus pyogenes* are sensitive to amicoumacin A at a level similar to *S. aureus* strains. These results indicated that amicoumacin A is highly effective against gram-positive cocci including important pathogens.

### Effect of amicoumacin A on the growth of *S. aureus* COL

**Figure 2:**
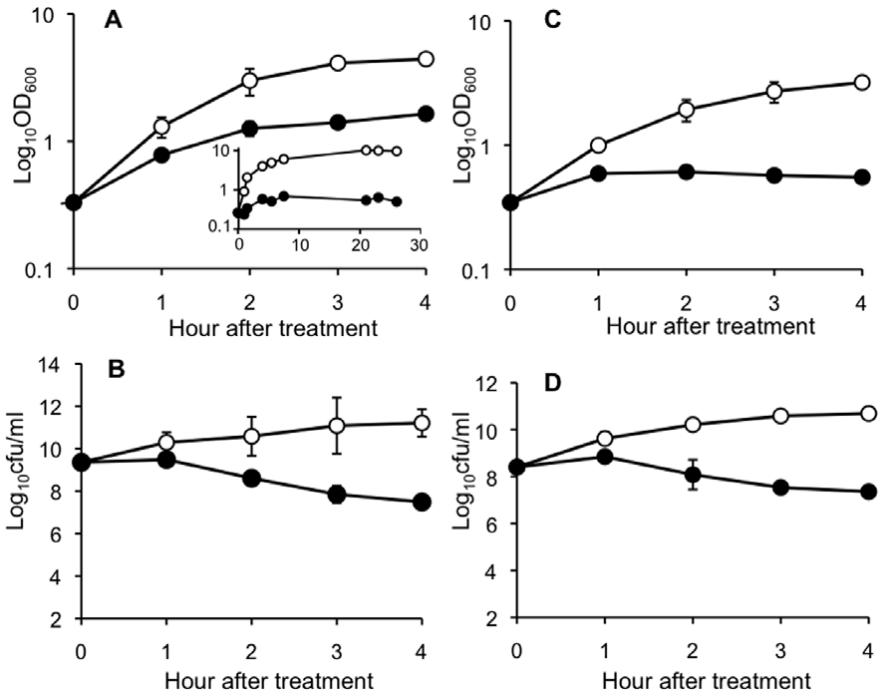
Effect of amicoumacin A (A and B) and vancomycin (C and D) on *S. aureus* COL. (A) COL was grown in TSB and exponential growth phase cultures were divided into two flasks. Amicoumacin-A-treated (closed circles) and -untreated cultures (open circles) were further incubated and the optical density at 600 nm was measured at hourly intervals. Effect of inhibitory levels of amicoumacin A during longer cultivation is shown in the insert of Figure 2A. (B) The cultures in (A) were used to examine viable cell counts by plating diluted cells on TSB agar. (C) COL was treated with 10 µg/ml of vancomycin and growth was monitored as described in (A). (D) The cultures in (C) were used to examine viable cell counts. The data are the average of three independent experiments with standard deviations.

The effect of amicoumacin A on the growth and survival of *S. aureus* COL was examined. As shown in Figure 2, the growth was inhibited after treatment with amicoumacin A and there was a significant ∼3-log_10_ decrease in the number of viable *S. aureus* cells. These effects on the growth and survival are similar to those by a bactericidal antibiotic vancomycin (Fig. 2), indicating that amicoumacin A is a bactericidal antibiotic as reported in *H. pylori* [16].

### Changes in global transcription of *S. aureus* COL in response to amicoumacin A

Transcriptome analysis has been used to identify changes in gene expression in response to antibiotic treatment (reviewed in [24]). Samples were taken at 0, 10, and 40 min (t_0_, t_10_, and t_40_, respectively) after COL was treated with amicoumacin A and RNA was purified as described in Materials and Methods. Microarray experiments were carried out on three independent biological replicates. The results are included as supplemental information (Table S1, all genes with a minimum 2.5-fold up or down change) and Table 1 shows a list of selected genes that are upregulated more than 3-fold at 10 min after the treatment compared to unexposed control cells.

**Table 1:** Selected genes induced 3-fold or above by amicoumacin A.

COL ORF	Fold induction		Gene product		
	T10	T40			
**Cell envelope**					
SACOL0248^a^	14.0	1.4	LrgB, antiholin		
SACOL0247^a^	8.6	1.0	LrgA, murein hydrolase regulator	−	
SACOL2197	9.2	11.9	Surface protein	+	σ^B^
SACOL2557	4.3	2.7	N-acetylmuramoyl-L-alanine amidase		
SACOL1062	3.9	4.0	Atl, bifunctional autolysin	+	σ^B^
SACOL0671^b^	3.9	2.2	α/β-fold family hydrolase	+	σ^B^
SACOL2597	3.5	1.4	α/β-fold family hydrolase	+	σ^B^
SACOL2019	3.8	3.3	SdrH protein	−	
SACOL2434	3.0	3.2	cell surface polysaccharide synthesis	+	σ^B^
**Transporter**					
SACOL2138^c^	6.7	7.8	Cation efflux family protein		
SACOL2136^c^	4.1	1.4	Hypothetical		σ^B^
SACOL0264	4.0	4.5	ABC transporter		
SACOL2176	3.8	2.2	OpuD2, osmoprotectant transporter	+	σ^B^
SACOL0681^d^	3.3	3.5	monovalent cation/H^+^ antiporter C		σ^B^
SACOL0679^d^	3.1	2.8	monovalent cation/H^+^ antiporter A		σ^B^
SACOL0682^d^	3.0	3.1	monovalent cation/H^+^ antiporter D		σ^B^
SACOL0680^d^	3.0	3.1	monovalent cation/H^+^ antiporter B		σ^B^
SACOL1422	3.1	3.7	phosphate ABC transporter		
**Virulence**					
SACOL2295	5.5	7.2	Staphyloxanthin biosynthesis		
SACOL0136^e^	3.8	2.7	Cap5A, capsular polysaccharide biosynthesis		σ^B^
SACOL0137^e^	3.0	2.3	Cap5B, capsular polysaccharide biosynthesis		σ^B^
SACOL0138^e^	3.4	2.0	Cap5C, capsular polysaccharide biosynthesis		σ^B^
SACOL0672^b^	3.4	2.0	SarA, staphylococcal accessory regulator	+	
SACOL0856	3.3	3.0	clumping factor	+	σ^B^
SACOL2511	3.0	5.4	fibronectin-binding protein		

Superscript alphabet indicates genes in the same operon. + and − arrows show genes induced or repressed in response to linoleic acid based on [25]. σ^B^-controlled genes are marked.

In total, we identified 263 upregulated and 282 downregulated genes.

The overall transcriptome results indicated that genes involved in diverse biological processes are upregulated, which is similar to the transcriptome in response to unsaturated long chain free fatty acids [25]. Particularly intriguing is that 46 genes were co-upregulated and 27 co-downregulated by amicoumacin A and linoleic acid. Of the 46 co-upregulated genes, 30 belong to the *S. aureus* σ^B^-regulon [26], [27]. σ^B^ is an alternative σ factor functioning in general stress response, the mechanism of which has been extensively studied in *B. subtilis* (reviewed in [28], [29]). A previous study suggested that σ^B^ of *S. aureus* functions in more basic cell physiology compared to the *B. subtilis* counterpart [27]. The σ^B^-controlled genes upregulated by amicoumacin A were either transiently upregulated at t_10_ only or were upregulated at t_10_ and t_40_. We examined whether the *sigB* mutation leads to a higher susceptibility to amicoumacin A; however, the growth curve assay did not show any significant effect of the *sigB* mutation on amicoumacin A susceptibility (data not shown).

An annotation-based grouping of amicoumacin A regulated genes highlighted a particularly high number of induced genes with cell envelope and transport related processes. In total, 21 genes with cell envelope associated functions were upregulated including *lrgA* (SACOL0247) and *lrgB* (SACOL0248), which are among the most highly induced genes (Table 1). The *lrgA* gene and its homologous gene *cidA* are known to regulate murein hydrolase activity and affect sensitivity to penicillin [30], [31]. CidA is a holin-like protein that positively controls murein hydrolase activity and penicillin-induced killing, which is antagonized by LrgA, an anti-holin [30], [31]. Consistent with the notion, *cidA* transcription is regulated oppositely from *lrgAB* in response to amicoumacin A. The microarray result showed that *cidA* is downregulated 1.9-fold at 10 min, whereas expression of the downstream *cidB* and *cidC* gene is upregulated 2.8-fold and 4.2-fold, respectively, which was confirmed by Northern blot analysis (data not shown).

In addition, transcription of three peptidoglycan hydrolases (*atl*, *lytM* and *sceD*) was strongly upregulated in amicoumacin-A-treated cells. Transcription of *atl* encoding the major autolysin of *S. aureus* was about 4-fold induced at t_10_ and t_40_, while *sceD* encoding a lytic transglycosylase [32] was 2-fold induced at t_10_ and over 10-fold at t_40_. Similar to *sceD*, transcription of *lytM*, a Gly-Gly endopeptidase [33], was 4-fold induced at t_40_. Another group of upregulated genes encode proteins that function in cell wall and surface polysaccharides synthesis and turnover, which include a penicillin-binding protein (SACOL1490), MurG (SACOL1453), a predicted glycosyl-transferase (SACOL2578), and N-acetylmuramoyl-L-alanine amidase (SACOL1825) [34]. In addition, over 46 genes encoding proteins with functions in membrane transport were upregulated.

### Validation of microarray results

**Figure 3:**
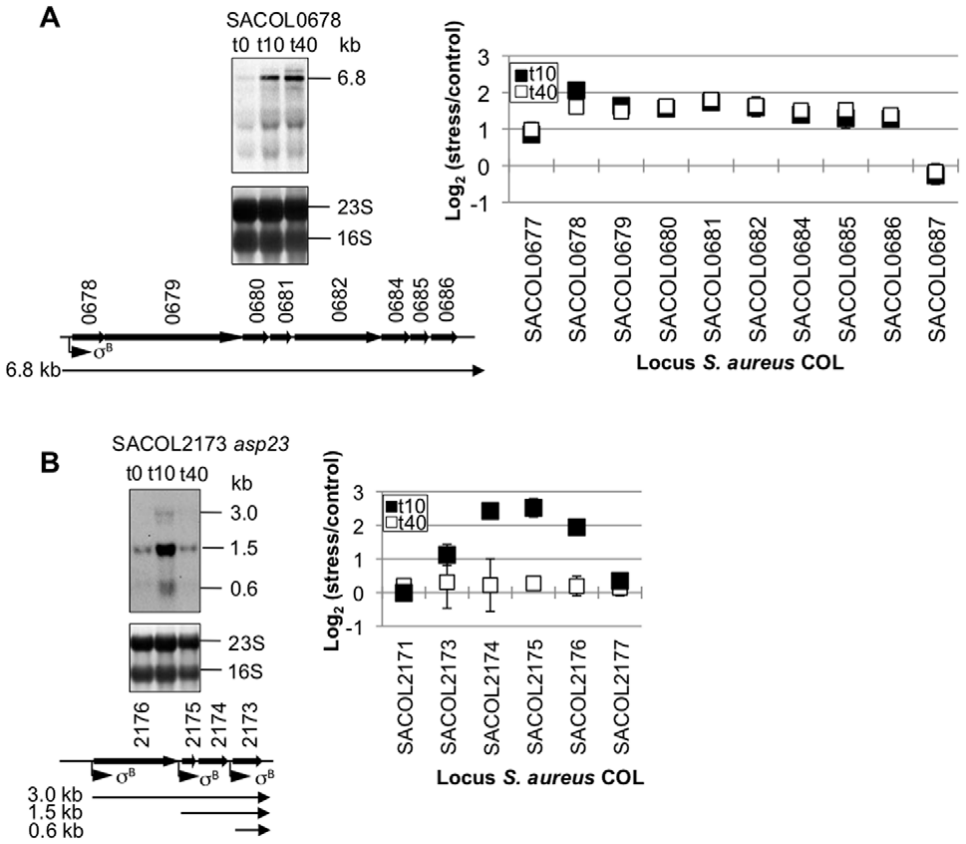
Northern blot analyses for SACOL0678 (A) and SACOL2176 (B) operon. Total RNA was isolated from *S. aureus* COL at 0 (t_0_), 10 (t_10_), and 40 (t_40_) min after the addition of amicoumacin A. 10 µg of total RNA isolated from each culture was separated in a formaldehyde-agarose gel and the RNA-blotted membrane was hybridized with SACOL0678- or SACOL2173(*asp23*)-specific digoxigenin-labeled probes. The sizes of the transcripts were determined by comparison to an RNA ladder on the same gel. The corresponding stained gels are shown underneath each blot. Schematic views of the gene loci based on NCBI COL genome site are shown with predicted transcripts. σ^B^ indicates locations of σ^B^-controlled promoters. Microarray results of each operon's genes are summarized in the right panel of the corresponding Northern blot gels. Closed squares and open squares show samples taken at t_10_ and t_40_, respectively. The average of triplicates and standard deviations are indicated.

Northern blot analysis was carried out with two independently isolated RNA samples to validate the microarray results. Five operons and one gene were randomly chosen and the results of two operons are shown in Figure 3. The operon of eight genes (SACOL0678 to SACOL0686) encodes a phage integrase family protein (SACOL0678) and monovalent cation/H^+^ antiporter subunits (SACOL0679 to 0686). A previous study showed that transcription of these genes is σ^B^-dependent [27]. Based on the microarray result, all eight genes were induced at t_10_ and the increased level of transcription was sustained at similar levels at t_40_ (Table 1 and Table S1). In the Northern blot experiment, a low level of 6.8 kb transcript was detected in untreated cells and the transcript was highly elevated at t_10_ and t_40_ (Figure 3A). This result confirmed that the eight genes constitute an operon and that amicoumacin A upregulates the operon transcription as shown by the microarray hybridization result. Transcription of another operon starting from SACOL2176 was also upregulated at t_10_ in the microarray results but the transcription decreased to the untreated level at t_40_ (Table 1 and Table S1). SACOL2176 encodes an osmoprotectant transporter, SACOL2175 and SACOL2174 encode a protein of unknown function and a membrane protein, respectively, and SACOL2173 is *asp23* that codes for alkaline shock protein 23 [35]. The Northern blot analysis detected three transcripts of 3.0 kb, 1.5 kb, and 0.6 kb, all of which increase at t_10_ but not at t_40_ (Figure 3B). The sizes of the transcripts correspond to the predicted transcripts initiated at the three σ^B^-dependent promoters. In a similar way, we also validated the microarray result of SACOL0673-0672-0671, SACOL2596-2597, SACOL02554.1-2554-2553, and SACOL1062 (data not shown).

### Effect of amicoumacin A on autolysis

**Figure 4:**
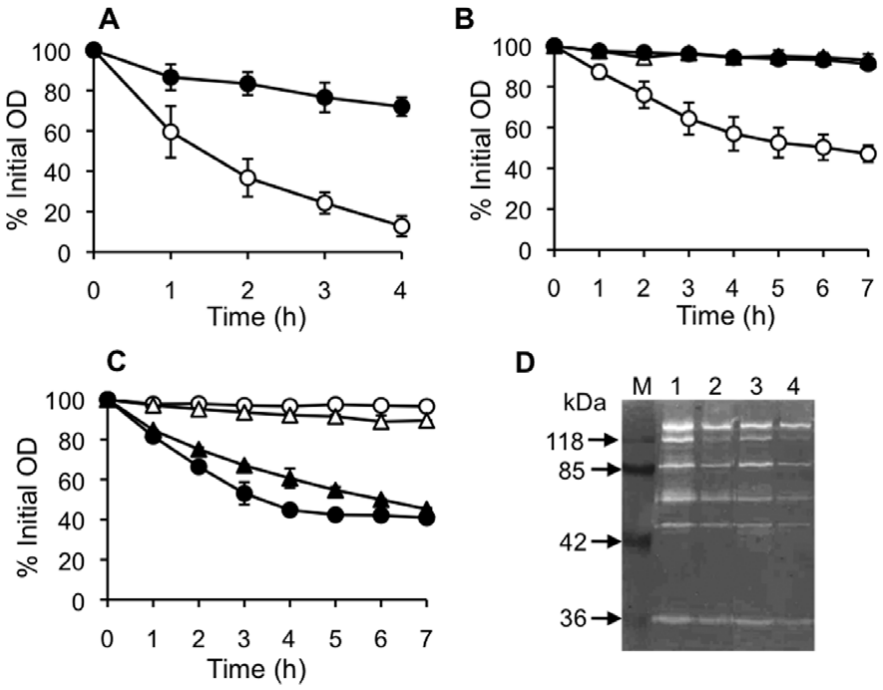
Effect of amicoumacin A on autolysis. (A) Effect on whole cell autolysis. Cultures grown in the absence (open circles) and the presence (closed circles) of amicoumacin A were washed and suspended in autolysis buffer to an initial OD_600_ of around 1.0 and autolysis was monitored as decline in OD_600_. (B) Quantitative assay of murein hydrolase activity against cell wall. An equal amount (90 µg protein) of extracellular proteins were added to cell wall purified from amicoumacin-A-untreated cells and OD_600_ was monitored as described in Materials and Methods. Symbols: open circles, extracellular proteins from untreated cells; closed circles, extracellular proteins from amicoumacin-A-treated cells; open triangles, 10 mM Tris-HCl (pH 7.5). (C) Susceptibility assay of purified cell wall to murein hydrolase. Ninety µg of extracellular proteins from amicoumacin-A-untreated cells were added to cell wall purified from amicoumacin-A-treated and -untreated cells and OD_600_ was monitored. Symbols: open circles, cell wall from untreated cells with 10 mM Tris-HCl (pH 7.5); closed circles, cell wall from untreated cells with extracellular proteins; open triangles, cell wall from amicoumacin-A-treated cells with 10 mM Tris-HCl (pH 7.5); closed triangles, cell wall from amicoumacin-A-treated cells with extracellular proteins. (D) Zymographic analysis of murein hydrolase activity from amicoumacin-A-treated and -untreated cells against purified *S. aureus* COL cell wall. Autolytic extracts were prepared and assayed by electrophoresis on an SDS-polyacrylamide gel (10%) containing 1 mg/ml purified cell wall as described in Materials and Methods. Lanes: M, prestained molecular weight markers; 1, cell-wall-associated proteins from untreated cells; 2, cell-wall-associated proteins from amicoumacin-A-treated cells; 3, extracellular proteins from untreated cells; 4, extracellular proteins from amicoumacin-A-treated cells.

The transcriptome results showed that *lrgA* transcription is highly upregulated and *cidA* transcription is downregulated by amicoumacin A. It has been shown that the *cidA* mutation reduces murein hydrolase activity [31], whereas the *lrgAB* mutation increases activity [30]. This result implied that autolysis might be reduced in response to amicoumacin A. On the other hand, the major autolysin gene *atl* was upregulated by amicoumacin A treatment (Table 1), suggesting that amicoumacin A might cause increased autolysis. To determine which possibility is correct, we investigated whether and how amicoumacin A affects autolysis. Figure 4A showed that amicoumacin-A-treated cells are more resistant to Triton X-100-induced lysis than untreated cells. Therefore, increased transcription of *atl* did not result in higher autolysis, which is consistent with the model that LrgA/CidA system controls autolysin activity at the post-transcriptional level [36].

To distinguish whether the reduced autolysis is due to alterations of cell wall or to decreased activity of autolysin, purified cell wall and extracellular autolysin were prepared from cells grown in the presence and absence of amicoumacin A. Previous work showed that overexpression of *lrgAB* in *S. aureus* leads to decreased extracellular murein hydrolase activity [30]. We first conducted an experiment designed to determine if amicoumacin A inhibits autolysis by reducing extracellular autolysin activity. Purified cell wall from *S. aureus* COL was incubated with an equal amount of extracellular proteins from untreated or amicoumacin-A-treated cells (Figure 4B). No significant decrease in OD_600_ was detected in the sample of purified cell wall alone, confirming that it has no intrinsic autolysin activity. The addition of extracellular proteins from untreated cells hydrolyzed over 50% of cell wall after 7 h of incubation, whereas proteins from treated cells did not hydrolyze purified cell wall, indicating that amicoumacin A reduces murein hydrolase activity. In a reciprocal experiment, we demonstrated that cell wall purified from strain COL grown in the presence of amicoumacin A was susceptible to extracellular autolysin from amicoumacin-A-untreated cells, although the rate of hydrolysis was slightly slower than cell wall prepared from untreated cells (Figure 4C). These results clearly showed that reduced autolysis of *S. aureus* grown in the presence of amicoumacin A is due to the lower level and/or activity of autolysin.

We performed zymographic analysis to examine the autolysin profile (Figure 4D). Both cell-wall-associated and extracellular autolysin profiles were not significantly altered by amicoumacin A treatment, but overall activities were reduced by the treatment. Except for the 36 kDa protein identified as Aaa (autolysin/adhesion protein of *S. aureus*) [37], the other six bands likely represent differently processed forms of Atl [38], [39]. Among these Atl forms, the 113 kDa band, which corresponds to an intermediate generated from pro-Atl by cleavage of the pro-peptide, was particularly reduced in both cell wall and the extracellular fraction of cells treated by amicoumacin A.

### Isolation of mutants with decreased susceptibility towards amicoumacin A

**Figure 5:**
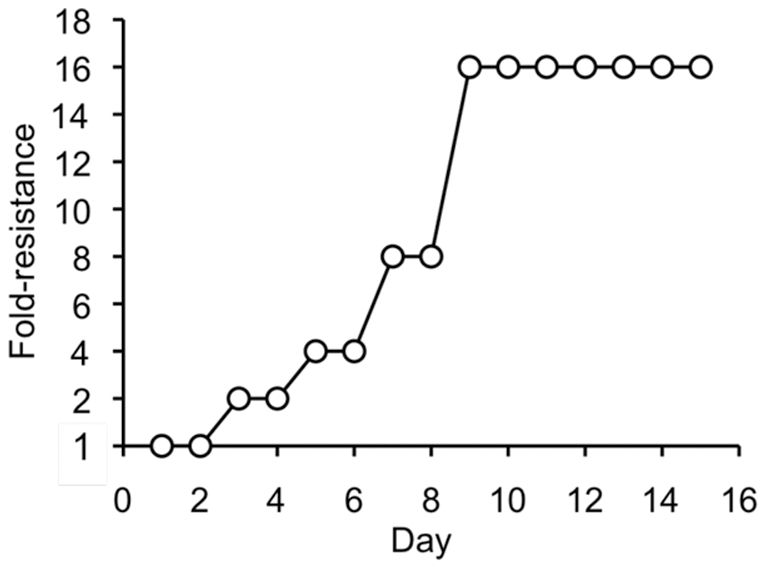
Serial passage experiments with *S. aureus* COL selected for increasing resistance to amicoumacin A. Each circle shows fold-resistance on each day.

As amicoumacin A is highly effective against MRSA, we investigated whether resistant mutants are associated with antibiotic selection pressure. Identification of mutations that lead to the amicoumacin A resistance might also provide clues to its mechanism of action. We isolated strains with decreased susceptibility to amicoumacin A using a serial passage of COL in the presence of the compound. COL was able to grow in the presence of successively higher concentrations of amicoumacin A over time. The resistance increased 2-fold every two days. Although we were able to obtain an isolate with 16-fold higher resistance against amicoumacin A as compared to the wild-type strain, further incubation up to 15 days did not increase resistance (Figure 5).

### Determination of the mutations associated with increased resistance to amicoumacin A

Whole-genome sequencing becomes a powerful tool for identifying genomic variation and was applied to track multidrug resistance in *S. aureus* recovered periodically from a patient undergoing chemotherapy with vancomycin [40]. We used the approach to identify genetic changes present in the amicoumacin A-resistant COL strains. Genome libraries were constructed from three resistant strains, namely SA-1, SA-3, and SA-6 that exhibit 2-fold, 8-fold, and 16-fold increase in resistance, respectively. The sequencing results uncovered a set of genes with varying numbers of single nucleotide polymorphisms (SNPs) in the resistant mutants (Table 2). The results revealed that more mutations were found in parallel with increasing levels of resistance. Among the mutations, SACOL0187 (in SA-1) and *oppB* (in SA-3) encode ABC-transporters. Both SA-3 and SA-6 contain missense mutations in *fusA* that encodes translation factor EF-G. Interestingly, the EF-G amino acid substitutions occur at adjacent glycine residues. The *fusA* gene has been known as the site of mutations that confer fusidic acid resistance in *Salmonella enterica* serovar Typhimurium [41] and *S. aureus* [42], [43]. Fusidic acid is a steroid-like antibiotic clinically used since the 1960s. Mutations that are responsible for fusidic acid resistance occur in domains I, III, and V of EF-G, as well as in domain II with less frequency, whereas the mutations found in the amicoumacin A-resistant isolates reside in domain IV. The *ksgA* mutation is the only nonsense mutation among the identified point mutations. The *ksgA* gene encodes the methyltransferase for two adjacent adenosines in 16 S rRNA. Kasugamycin resistance was shown to be associated with the loss of the methylation [44]. Other mutations were identified in *dnaG* encoding primase, *lacD* encoding tagatose 1,6-bisphosphate aldolase, and SACOL0611, a putative glycosyl transferase gene.

**Table 2:** Codon substitution mutations found in amicoumacin-A-resistant COL.

Isolate	Fold-resistance	Mutated gene (mutation)	Function
SA-1	2	SACOL0187 (D343H)	RGD-containing lipoprotein
SA-3	8	*fusA* (G530V)	Elongation factor G
		*oppB* (I263F)	Oligopeptide permease
		*dnaG* (R190H)	DNA primase
SA-6	16	*fusA* (G531S)	Elongation factor G
		*ksgA* (L260Stp)	16 S rRNA methyl transferase
		*lacD* (G86R)	tagatose-1,6-bisphosphate aldolase
		SACOL0611 (S472P)	Glycosyl transferase

### Construction of the *ksgA* knockout mutation and amicoumacin A resistance

To evaluate the role and impact of the *ksgA* mutation in amicoumacin A resistance, the same mutation was re-created in COL using plasmid pMAD with a temperature-sensitive replicon. The mutant allele (L260stp) of the *ksgA* gene that lacks the start codon was amplified by PCR and the product was cloned into pMAD. The PCR product was designed to position the mutation at the 3′-end of the insert DNA, thus maximizing the chance of homologous recombination at a region located the 5′ to the site of the mutation. A single crossover recombination of the resultant plasmid into COL chromosomal DNA thus results in the mutant *ksgA* gene expressed from the native promoter and inactivation of the wild-type *ksgA* gene due to the loss of its promoter and deletion of the 5′-end of the gene. The integration of the plasmid in the predicted manner was confirmed by PCR and sequence analysis of the *ksgA* region of the resultant strain, SA-8. The MIC of amicoumacin A increased 2-fold compared to the parent strain, confirming that the nonsense *ksgA* allele is responsible for a low level of resistance towards amicoumacin A. As expected, SA-8, like SA-6, exhibited increased kasugamycin resistance compared to the wild-type strain (data not shown).

### Overexpression of *fusA* genes with mutant alleles in *S. aureus* COL

As described above, the *fusA* mutations found in two amicoumacin A-resistant strains locate in domain IV where no fusidic acid-resistant mutation has previously been isolated. We also found that the SA-3 and SA-6 *fusA* mutants are as sensitive to fusidic acid as the wild-type strain (MIC 0.08 µg/ml). In order to further characterize antibiotic-resistant phenotypes of *fusA* mutations, we isolated a fusidic acid-resistant mutant (SA-7) by the multiple passage method as described in Materials and Methods. The MIC of SA-7 for fusidic acid was 12.5 µg/ml, which is 156-fold higher than that of the wild-type strain, whereas SA-7 exhibited the same MIC as the wild-type COL towards amicoumacin A. Sequence analysis of *fusA* amplified from the SA-7 chromosomal DNA identified a point mutation of R464H in domain III, which is identical to the mutation identified in a fusidic acid-resistant *S. aureus* with the small-colony-variant phenotype [43].

We next checked whether SA-11, the wild-type COL strain carrying the mutant allele (R464H) of *fusA* on plasmid pDG148, confers resistance to fusidic acid. The MIC of this strain for fusidic acid was around 6.25 µg/ml irrespective of IPTG addition, probably because the control of the vector IPTG-inducible P*spac* promoter is leaky. In contrast, the MIC of SA-11 for amicoumacin A was identical to that of the wild-type strain. SA-9 and SA-10 that express *fusA*(G530V) and *fusA*(G531S), respectively, on the multi-copy plasmid were sensitive to fusidic acid but exhibited 2-fold higher resistance to amicoumacin A as compared to the wild-type strain. These results confirmed that the *fusA*(G530V) and *fusA*(G531S) mutations cause amicoumacin A resistance and the *fusA*(R464H) mutation causes fusidic acid resistance, but not *vice versa*.

## Discussion

Amicoumacin A possesses strong bactericidal activity against MRSA. Xenocoumacin 1, another isocoumarin antibiotic, shares an identical dihydroisocoumarin moiety with amicoumacin A, but has a slightly different hydroxy amino acid side chain (Figure 1). Both compounds exhibit potent activity against gram-positive bacteria, but only xenocoumacin 1 exhibits activity against selected gram-negative bacteria, such as some *E. coli* strains [14]. Conversely, amicoumacin A can kill multi-resistant *S. aureus*, whereas xenocoumacin I has no effect on the organism [14]. This difference in spectra of activity might be due to the difference in the hydroxy amino acid side chains of the two molecules. In fact, it has been previously demonstrated that the amide at the terminal end of the side chain in amicoumacin A is essential for its activity against MRSA [19].

In this study, we aimed at elucidating the mechanism behind the strong bactericidal activity of amicoumacin A against MRSA. In hope of identifying the target of amicoumacin A, we isolated amicoumacin A resistant strains using the multiple passage method.

Two different alleles of *fusA* were found to cause amicoumacin A resistance, while other alleles lead to fusidic acid resistance as shown here and in previous work. Fusidic acid binds to an EF-G/ribosome complex and inhibits EF-G turnover. The crystal structure of *Thermus thermophilus* EF-G revealed that domains I and II are homologous to elongation factor Tu and domains III and V show structural similarities to ribosomal proteins [45]. Domain IV was shown to protrude from the main body of the protein. This could explain why no cross-resistance was observed between fusidic acid-resistant mutations found in all domains except domain IV and amicoumacin A-resistant mutations in domain IV. The high resistance to fusidic acid caused by a single mutation in *fusA* is attributed to the fact that EF-G is the direct target of fusidic acid [46]. On the contrary, amicoumacin A resistance caused by the *fusA* mutations or the *ksgA* mutation is only 2-fold. In addition, the resistance increased every 2-fold in parallel with increased numbers of mutations in such a way that 2-, 8-, and 16-fold increase in MIC was observed in the strains carrying 1, 3, and 4 point mutations. These results suggest that the direct target of amicoumacin A is neither EF-G nor proteins encoded by the other genes, the mutations of which were identified by genome-wide sequencing.

As described above, *lrgAB* transcription was most highly induced by amicoumacin A. The proton ionophore carbomyl cyanide *m*-chlorophenylhydrazone (CCCP) was known to induce *lrgAB* expression. Since valinomycin, which dissipates Δψ, but not nigericin that depletes ΔpH across the membrane, has a similar effect with CCCP on *lrg* expression, the authors concluded that *lrgAB* is upregulated in response to a collapse of Δψ [47]. As this induction of *lrgAB* transcription requires the LytSR two-component signal transduction system, the LytS kinase might respond to a collapse of Δψ [47]. Daptomycin induces genes including *lrgAB* that are induced by CCCP, hence it was proposed that a part of the mechanism of action of daptomycin is membrane depolarization [48], which is consistent with an earlier study on potassium release assays [18]. Given transcription of *lrgAB* is highly induced by amicoumacin A, it might be possible that membrane depolarization is at least a part of the mechanism of action.

In this study, we showed that autolysis is highly reduced in cells grown in the presence of amicoumacin A, which is mainly caused by decreased activity of murein hydrolase, very likely Atl. A similar inhibition of autolysis due to reduced murein hydrolase activity was recently reported in *S. aureus* treated with Magnolol (5, 5′-diallyl-2, 2′-dihydroxybiphenyl) [49]. In this case, transcription of *atl* and *cidA* was reduced and *lrgAB* transcription was highly induced after Magnolol treatment. In the case of amicoumacin A, *atl* transcription was upregulated and Atl activity was reduced. There is a precedent for this paradoxical observation. A teicoplanin-resistant MRSA isolate exhibited an autolysis-deficient phenotype, although expression of major autolytic genes (*atl*, *lytM*, and *lytN*) was not changed [50]. As the resistant strain displayed decreased extracellular murein hydrolase activity, the authors suggested post-transcriptional regulation of hydrolase activity, the same conclusion of our study. However, transcription analysis of *cidABC* and *lrgAB* in the teicoplanin-resistant strain ruled out the possibility of a role for CidA in post-transcriptional control of murein hydrolase activity. The major murein hydrolase Atl is involved in peptidoglycan turnover, daughter cell separation after cell division [51], biofilm formation [52], and penicillin-induced lysis [53]. Given the roles of Atl in diverse cellular function, its activity could be tightly controlled through multiple mechanisms. Future studies are needed to uncover whether amicoumacin A affects autolysin activity through the LytS-Lrg-Cid pathway.

## Materials and Methods

### Bacterial strains and media

All of the strains used in this study are listed in Table 3. *Bacillus* and *Escherichia coli* were cultured in 2×YT and methicillin-sensitive *S. aureus* (MSSA) and MRSA strains were routinely propagated in tryptic soy broth (TSB) (Cellgro, Manassas, VA). Half-diluted artificial seawater contains 13.75 g NaCl, 5.3 g MgCl_2_⋅6H_2_O, 1.95 g MgSO_4_⋅7H_2_O, 0.5 g KCl, 0.331 g CaCl_2_⋅2H_2_O, and 0.914 mg FeSO_4_⋅7H_2_O per liter. Antibiotic concentrations used are; ampicillin, 50 µg/ml; erythromycin, 10 µg/ml; kanamycin, 100 µg/ml (for RN4220) and 20 µg/ml (for COL).

**Table 3:** Plasmids and bacterial strains.

	Plasmid	
pDG148	multi-copy shuttle vector, kanamycin resistance	[57]
pMAD	integration plasmid with temperature-sensitive replicon	[56]
pALG26	pMAD with *ksgA*(L260stp)	
pALG43	pDG148 with *fusA*(G530V)	
pALG44	pDG148 with *fusA*(G531S)	
pALG45	pDG148 with *fusA*(R464H)	

	Strain	
*Bacillus subtilis* JH642	Laboratory stock	James Hoch
*Bacillus pumilus* C9	Isolated from the Columbia River Estuary	
SAFR032		Kasthuri Venkateswaran
8A1	Bacillus genetic stock center	
8A2	Bacillus genetic stock center	
8A3	Bacillus genetic stock center	
8A4	Bacillus genetic stock center	
8E2	Bacillus genetic stock center	
8E3	Bacillus genetic stock center	
14A1	Bacillus genetic stock center	
*Bacillus anthracis* Sterne 7702	Laboratory stock	Terri Koehler
*Bacillus cereus* ATCC10987	Bacillus genetic stock center	
*Bacillus thuringiensis* NRRL-B4039	Bacillus genetic stock center	
*Listeria monocytogenes* F4244	Laboratory stock	
*Staphylococcus aureus*		
RN4220 (MSSA)	Laboratory stock	Barry Hurlburt
RN6390 (MSSA)	Laboratory stock	Barry Hurlburt
COL (HA-MRSA)		Ambrose Cheung
SA-1	amicoumacin A resistant	
SA-3	amicoumacin A resistant	
SA-6	amicoumacin A resistant	
SA-7	fusidic acid resistant	
SA-8	*ksgA*::pALG26	
SA-9	COL with pDG148 carrying *fusA*(G530V)	
SA-10	COL with pDG148 carrying *fusA*(G531S)	
SA-11	COL with pDG148 carrying *fusA*(R464H)	
N315 (HA-MRSA)		Ambrose Cheung
Mu50 (HA-MRSA) VISA		Ambrose Cheung
USA300 (CA-MRSA)		Ambrose Cheung
USA400 (CA-MRSA)		Ambrose Cheung
*Staphylococcus carnosus*/pCX15	Laboratory stock	Wolfgang Schumann
*Streptococcus pyogenes* MGAS5005	Laboratory stock	June Scott
*Escherichia coli* DH5α	Laboratory stock	
*Pseudomonas aeruginosa* PA01	Laboratory stock	

### Identification of *Bacillus pumilus* C9 that produces amicoumacin A

Water samples were collected from the estuary turbidity maximum of the North Channel in the Columbia River Estuary on June 14, 2007 (cruise data link http://www.stccmop.org/node/510). No specific permissions were required for the described field studies. The water samples were incubated at 28°C for 8 days and plated on half-diluted artificial seawater containing 0.5% peptone, 0.1% yeast extract (SPY), and 1.5% agar. After incubation at 28°C for 3 to 4 days, each bacterium was purified by isolating a single colony. Isolated bacterium was grown on SPY agar for 2 to 3 days depending on growth and antimicrobial activity was determined by overlaying *S. aureus* RN6390 in 7 ml brain heart infusion (BHI) (Becton Dickinson, Franklin Lakes, NJ) with 0.7% soft agar. After incubation at 37°C overnight, antimicrobial activity was detected as a lysis zone of RN6390 around a colony of testing bacteria.

### Overlay assay to determine anti-microbial spectrum of amicoumacin A

*B. pumilus* C9 was streaked onto a small piece (1×1 cm) of 0.22 µm Millipore filter placed on LB agar plate and incubated at 37°C overnight. The filters were removed and susceptibility to amicoumacin A was determined by overlaying cultures of testing strains in 7 ml of BHI or 2×YT with soft agar. The plates were incubated at 37°C for 5 h to overnight.

### Isolation of the anti-MRSA substance and purification to homogeneity

A fresh colony of *B. pumilus* C9 was used to inoculate LB broth and was incubated by shaking (150 rpm) at 30°C for 24 h. The culture supernatant was passed through an Amicon® ultra filtration membrane (MW 3,000 cutoff, Millipore, Billerica, MA) and the filtrate was lyophilized. The lyophilized material was extracted with methanol and the dried methanol extract was dissolved in 20 mM Tris-HCl (pH 7.0) before applying to a Hi-Q column to remove impurities. The column was run with 20 mM Tris-HCl (pH 7.0) at a flow rate of 2 ml/min. The active fractions were pooled and lyophilized for further purification by reverse phase high performance liquid chromatography (RP-HPLC). A C18 semi-preparative column (Vydac 218TP510, 300 Å pore diameter, 250 mm×10 mm, 5 µm particle diameter) was developed with a gradient of 5% acetonitrile/0.1% trifluoroacetic acid (TFA) to 50% acetonitrile/0.1% TFA from 5 to 42 min using a 3 ml/min flow rate. The active peak was identified at 29.56 min, which corresponds to an acetonitrile concentration of 36%. This fraction was lyophilized prior to analysis by mass spectrometry and nuclear magnetic resonance (NMR) spectroscopy.

### Mass spectrometry analysis

The purified anti-MRSA substance was analyzed by nano-electrospray ionization (nanoESI) Fourier transform ion cyclotron resonance (FTICR) mass spectrometry. The Bruker 9.4T Apex-Qe FTICR (Bruker Daltonics, Billerica, MA) was calibrated externally using polyethylene glycol. The sample was dissolved in 50% acetonitrile/0.1% formic acid for injection into the nanoESI.

### NMR analysis

The purified anti-MRSA substance (200 µg) was dissolved in 300 µl of deuterium oxide (D_2_O, 99.96% D, Cambridge Isotopes Laboratories, Cambridge, MA) that also contained 100 µM 2,2-dimethyl-2-silapentane-5-sulfonate sodium salt (DSS). The sample was pipetted into a D_2_O-matched Shigemi NMR tube (Shigemi Co., Ltd., Tokyo, Japan) for NMR analysis. NMR experiments were performed on the sample using a Varian Inova 800-MHz spectrometer equipped with a triple resonance HCN probe and Z-axis pulsed field gradients.

### Partial purification of amicoumacin A

For routine purposes such as MIC and growth inhibitory assay experiments, partially purified amicoumacin A was used. *B. pumilus* C9 culture supernatant was extracted with one-fourth volume of n-butanol and shaking for 2 h at 200 rpm. The mixture was then poured into a separatory funnel and allowed to stand for another 2 h. The organic layer was collected and evaporated at 55°C overnight. The dried sample was dissolved in 10 mM Tris-HCl (pH 7.0) and passed through an Amicon ultra filtration membrane (MW 3,000 cutoff) and the filtrate was re-extracted with butanol. The dried sample was resuspended with 10 mM Tris-HCl (pH 7.0) and then passed through LH-20 Sephadex column with 1 ml/min flow rate. The active fractions were pooled and extracted with butanol. The evaporated sample was dissolved in 10 mM Tris-HCl (pH 7.0) and aliquots were frozen at −80°C until use.

### Growth profile and survival assay of *S. aureus* COL in the presence of amicoumacin A

A fresh colony of *S. aureus* was inoculated and cultured in TSB overnight at 37°C. Two hundred µl of the overnight culture was inoculated into a 250 ml flask containing 20 ml of TSB. The flask was incubated in a 37°C shaker until the OD_600_ reached 0.4–0.5. The culture was divided into two flasks, 50 µl of amicoumacin A or 10 mM Tris-HCl (pH 7.0) was added to each flask and incubation was continued at 37°C. The readings at OD_600_ were recorded at every 1 h. In order to determine the cell survival profile in response to amicoumacin A, the cells were withdrawn at 0 h and every 1 h after the drug treatment, serially diluted, and plated onto TSB agar. Numbers of colonies were counted after overnight incubation at 37°C.

### MIC assays

MICs for *S. aureus* COL were determined by broth microdilution assays in 96 well microtiter plates. Fifty µl of 2-fold serial dilutions of amicoumacin A were mixed with an equal volume of 1∶100 diluted overnight COL cultures in TSB. Control wells without drug were also included. The microtiter plate was incubated at 37°C for 24 h.

### Transcriptome analysis

A fresh colony of COL was used to inoculate 2 ml of BHI. The overnight culture (0.5 ml) was transferred to 50 ml BHI and incubated at 37°C until OD_600_≈0.5 (t_0_), when 20 ml of cultures were withdrawn into 10 ml of ice-cold killing buffer (20 mM sodium azide, 5 mM MgCl_2_, 20 mM Tris-HCl, pH 7.5). To the remaining cultures, amicoumacin A was added at the concentration leading to around 12% and 20% reduction of OD_600_ after 10 min and 40 min, respectively. At 10 min and 40 min (t_10_ and t_40_) after the addition of amicoumacin A, 10 to 15 ml of cultures were harvested by mixing a half volume of ice-cold killing buffer. Harvested cells were immediately centrifuged at 4°C for 5 min at 7,000× g and washed once with killing buffer before being frozen at −80°C. Each experiment was performed in biological triplicate. RNA was prepared from harvested cells using glass beads/phenol method as previously described [54] and DNA was removed using RNase-free RQ1 DNase (Promega, Madison, WI). The quality of RNA was checked by gel electrophoresis and an Agilent bioanalyzer.

The design and evaluation of the customized StaphChip oligoarray manufactured by Agilent Technologies (Palo Alto, CA, USA) used in this study has been described elsewhere [55]. Microarray analysis was employed as described previously [56]. In short, cDNA was synthesized from 10 µg of total RNA and labeled by incorporation of Cy5-dCTP or Cy3-dCTP (GE Healthcare, Little Chalfont, United Kingdom) by direct reverse-transcription using Superscript II (Invitrogen, Karlsruhe, Germany) and random hexamers (Promega Madison, WI) as primers. After denaturation of the RNA primer mix for 10 min at 70°C, cDNA synthesis was performed in a 50 µl reaction volume at 42°C for 60 min. The concentrations of enzyme and reagents in the reverse transcription reaction were as follows: Superscript II (400 units), Cy-dye (1.25 nmol), dATP, dGTP, dTTP (5 nmol each), dCTP (2.5 nmol), random hexamers (1.25 µg), DTT (0.01 M) and 1× first strand buffer. The Superscript II was heat-inactivated for 10 min at 70°C and template RNA degraded by incubation for 30 min at room temperature with *E. coli* RNase H (2 units) (Invitrogen, Karlsruhe, Germany). Labeled cDNA was purified with the CyScribe GFX Purification Kit following the instructions of the manufacturer (GE Healthcare, Little Chalfont, United Kingdom) and Cy-dye incorporation was analysed with a NanoDrop ND-1000 spectrophotometer (NanoDrop Technologies, Inc., Rockland, DE). Approximately 500 ng of each labelled cDNA corresponding to 3–6 pmol of incorporated dye were used in two-color pool-cDNA (Cy3) versus sample cDNA (Cy5) competitive hybridization experiments. For the pool-cDNA synthesis equal amounts of all RNA samples were mixed. The pool was used as common reference. For each time point (t_0_, t_10_, and t_40_), three biologically independent cDNAs were synthesized.

Hybridizations were done in a total sample volume of 40 µl for 17 h at 65°C at 10 rpm in a dedicated hybridization chamber (Agilent Technologies) and hybridization oven (Robbins Scientific, Sunnyvale, CA, USA). After hybridization, the slides were washed for 1 min at room temperature in wash buffer 1 followed by a 1-min washing step at 37°C with wash buffer 2. The slides were dried by submersion in acetonitrile for 30 sec. Blocking reagents, hybridization buffer (Gene Expression Hybridization Kit) and washing solutions (Gene Expression Wash Buffer Kit) were purchased from Agilent. Slides were scanned at a 5 µm resolution (Agilent Technologies Scanner) and fluorescence intensities were extracted and processed using the Feature Extraction™ software version 9.5.3.1 (Agilent Technologies). Local background-subtracted signals of both fluorescence channels were normalized with the linear LOWESS function. To test whether genes were differentially expressed in response to amicoumacin A, a Welch's t-test (α of <0.05) with a Bejamini and Hochberg FDR correction for multiple testing was calculated in Genespring (Agilent Technologies). Genes that passed the significance test for at least one time point (t_0_ versus t_10_, t_0_ versus t_40_ or t_10_ versus t_40_) and with a mean 2.5-fold up or 2.5-fold down regulation were considered differentially expressed. Grouping of genes based on expression profiles was calculated with the hierarchical clustering algorithm implemented in the multiple experiment viewer MeV [57]. The complete microarray data set is available at the NIH Gene Expression Omnibus (GEO) database under record number GSE31342.

### Northern blot analyses

Digoxigenin-labeled RNA probes were prepared by in vitro transcription with T7 RNA polymerase by using a PCR fragment (SACOL0678) or plasmid (*asp23*: pKSAP23 [58]) as template. The PCR fragment was generated from COL chromosomal DNA purified with a chromosomal DNA isolation kit (Promega, Madison, WI) and the respective oligonucleotides (SA0678_COL_F: 5′-ATGAATAA AGTAGAAGCGAT-3′ and SA0678_COL_RT7: 5′-CTAATACGACTCACTATAGGG AGACTATAATTGTA ATGAAATAT-3′). Northern blot analyses were carried out as previously described [59]. The digoxigenin-labeled RNA marker I (Roche, Indianapolis, IN) was used to calculate the sizes of the transcripts. The hybridization signals were detected using a Lumi-Imager (Roche Diagnostics, Mannheim, Germany) and analyzed using the software package Lumi-Analyst (Roche Diagnostics, Mannheim, Germany).

### Autolysis assay

Cultures at OD_600_ of around 0.3 were either treated with amicoumacin A for 45 min or left untreated. Harvested cells from each culture were washed with cold water and suspended at OD_600_ of 1.0 in 50 mM Tris-HCl (pH 7.5) with 0.05% Triton X-100. Autolysis was measured during incubation at 37°C with shaking as a decrease in optical density at OD_600_.

### Preparation of purified cell wall

Purified cell wall was prepared as described previously [60]. In short, cells harvested at OD_600_ of 0.6 were boiled for 30 min in 4% SDS and washed with water for 4 times. Cells were broken with glass beads on a Vortex mixer, followed by treatment with α-amylase (100 µg/ml), DNase (10 µg/ml), RNase (50 µg/ml), and trypsin (100 µg/ml). The enzymes were inactivated by boiling for 15 min in 1% SDS. Cell wall was collected by centrifugation and washed twice with water, once with 8 M LiCl, once with 100 mM EDTA, and twice with water. Acetone-washed pellet was resuspended in water and lyophilized.

### Crude autolytic enzyme extracts

Cells untreated or treated with amicoumacin A for 45 min were harvested by centrifugation, washed twice with 50 mM Tris-HCl (pH 7.5). After centrifugation, pellet was suspended in 50 mM Tris-HCl-2% SDS (at the concentration of 200OD_600_/ml). After centrifugation, supernatant was saved as cell-wall-associated autolysin. As for a source of extracellular autolysins, culture media were filtered through Millipore filter (0.22 µm) and concentrated around 40-fold using Amicon Ultra 3 K. The concentrated culture media were adjusted by standardizing by OD_600_ harvested cells. Protein concentration of concentrated media was measured by BioRad assay and extracellular protein concentrations were at 0.6 µg/ml in thus concentrated cultured media from untreated and treated cells.

### Cell wall hydrolysis *in vitro*

Purified cell wall isolated from *S. aureus* COL cultured in the absence and presence of amicoumacin A was suspended in 50 mM Tris-HCl (pH 7.5) to an OD_600_ of 0.7. Cell wall hydrolysis was initiated by addition of extracellular proteins (90 µg) prepared as described above. As a control, equal volume of 10 mM Tris-HCl (pH 7.5) was added. Activities of autolysins were monitored by a decline in OD_600_ during incubation at 37°C.

Detection of murein lytic activity was also carried out by zymographic analysis. Cell-wall-associated proteins prepared from 0.2 OD_600_ amicoumacin-A-treated or untreated cells (1 µl of 200OD_600_/ml cell wall extract) and 1.9 µg of extracellular proteins were separated in an SDS-10% polyacrylamide gel containing purified cell wall (1 mg/ml). After electrophoresis, the gel was rinsed with water and incubated overnight at 37°C in 25 mM Tris-HCl (pH 8.0) containing 1% Triton X-100. The gel was rinsed with water and stained with 0.1% methylene blue in 0.01% KOH.

### Isolation of mutants with decreased susceptibility to amicoumacin A and fusidic acid

Mutants were isolated by multiple passage methods through progressively increasing concentration of amicoumacin A or fusidic acid. Bacterial cultures that grew at the highest concentrations of the drug were used as an inoculum for the subsequent culture. Three amicoumacin A-resistant strains (SA-1, SA-3, and SA-6) and one fusidic acid-resistant strain (SA-7) were used for further studies.

### Genotypic characterization of amicoumacin A-resistant mutants

Comparative genome sequencing (CGS) was used to identify chromosomal mutations in amicoumacin A-resistant mutants of *S. aureus* COL, SA-1 (2-fold increase in MIC), SA-3 (8-fold), and SA-6 (16-fold). Genomic DNA was purified from the wild-type and mutant strains using Wizard® Genomic DNA Purification Kit (Promega, Madison, WI). DNA was sheared to the fragments of less than 800 bp using Nebulizer kit (Invitrogen, Carlsbad, CA). The fragments were blunt-ended using end-it DNA repair kit (Epicentre, Madison, WI) and “A-tailing” of the fragments was done using Klenow exo-minus and dATP. The adapters with different barcodes and an overhang-T were ligated to the A-tailed fragments using fast link kit (Epicentre, Madison, WI). The ligated products were loaded onto 2% agarose gels. A 400–450 bp range of DNA was cut from the gel to exclude the unligated adapters and eluted using gel extraction kit (Qiagen, Valencia, CA). PCR was carried out using primers DL139 (5′-AATGATACGGCGACCACCGAGATCTACACTCTTTCCCTACACGA-3′) and DL140 (5′-CAAGCAGAAGACGGCATACGAGATCGGTCTCGGCATTCCTGCTGA AC-3′) and PfuUltra™ II Fusion HS DNA polymerase (Stratagene, Santa Clara). The PCR fragments were cloned using TOPO blunt kit (Invitrogen, Carlsbad, CA) and 10 randomly chosen clones from each library were sequenced to check the quality of genomic DNA library. The library DNA was diluted to 20 nM and sent to the Genomics Core Facility at Tufts University School of Medicine for single-end sequencing in Illumina HiSeq 2000. The analysis was carried out using the wild-type COL genome as a control and later comparing with *S. aureus* COL genome available at NCBI (Accession no. NC_002951).

### Construction of *ksgA* mutants

The *ksgA* gene carrying the mutation (L260stop) was amplified using primers, ksgAFEcoRI (5′-GCTCGAATTCGGATAATAAAGATA TTGCAACACC-3′, *Eco*RI site is underlined) and ksgARBamHI (5′-GATCGGATCCGCCTCCATTGGCTTTCAG TACAATAC-3′, *Bam*HI site is underlined), and chromosomal DNA isolated from the SA-6 strain as a template. The PCR product digested with *Eco*RI and *Bam*HI was cloned into pMAD vector [61] digested with the same enzymes to generate pALG26. pALG26 was introduced into RN4220 by electroporation with selection for erythromycin resistance (Erm^r^) at 30°C. Bacteriophage φ11-mediated transduction was used to introduce pALG26 from the RN4220 transformant into COL by selecting transductants on TSB agar plates containing erythromycin and X-gal at 30°C. A single clone of the transductants was grown in TSB containing erythromycin at 30°C for 3–4 h and at 42°C for 6 h before plating on TSB agar plates containing erythromycin and X-gal. After incubation at 42°C, light blue colonies were obtained, which resulted from a single crossover recombination between the plasmid-born and the chromosomal *ksgA* genes. The recombinant strain, SA-8, was checked for resistance against kasugamycin and amicoumacin A.

### Overexpression of *fusA* carrying the mutant alleles in *S. aureus* COL

The wild-type and the *fusA* mutant (G530V, G531S, R464H) genes were amplified using primers fusASalIF (5′-GATCGTCGACATGGCTAGAGAATTTTCA-3′, *Sal*I site is underlined) and fusASphIR (5′-GATCGCATGCTTATTCACCTTTATTTTT C-3′, *Sph*I site is underlined) from chromosomal DNA isolated from SA-3, SA-6, and SA-7, respectively. Each PCR product was digested with *Sal*I and *Sph*I, and ligated with pDG148 [62] digested with the same enzymes to generate pALG43 (for G530V), pALG44 (for G531S), and pALG45 (for R464H). These plasmids were first transformed into RN4220, then introduced into COL by φ11-mediated transduction as described above. The resultant COL strains, SA-9 (G530V), SA-10 (G531S), and SA-11 (R464H), were examined for fusidic acid- and amicoumacin A-susceptibility by microdilution method.

## Supporting Information

Table S1 **Changes in global transcription of** ***S. aureus*** **COL in response to amicoumacin A.** Hierarchical clustering has been done to classify the regulated genes based on their expression profiles. Genes in clusters 1–4 were upregulated and those in clusters 5–8 were downregulated.
(PDF)

## References

[1] Chambers HF, DeLeo FR (2009). Waves of resistance: *Staphylococcus aureus* in the antibiotic era.. Nat Rev Microbiol.

[2] Fuda C, Suvorov M, Vakulenko SB, Mobashery S (2004). The basis for resistance to beta-lactam antibiotics by penicillin-binding protein 2a of methicillin-resistant *Staphylococcus aureus*.. J Biol Chem.

[3] Couto I, de Lencastre H, Severina E, Kloos W, Webster JA (1996). Ubiquitous presence of a *mecA* homologue in natural isolates of *Staphylococcus sciuri*.. Microb Drug Resist.

[4] Ito T, Katayama Y, Hiramatsu K (1999). Cloning and nucleotide sequence determination of the entire mec DNA of pre-methicillin-resistant *Staphylococcus aureus* N315.. Antimicrob Agents Chemother.

[5] Boucher HW, Corey GR (2008). Epidemiology of methicillin-resistant *Staphylococcus aureus*.. Clin Infect Dis.

[6] Hiramatsu K, Aritaka N, Hanaki H, Kawasaki S, Hosoda Y (1997). Dissemination in Japanese hospitals of strains of *Staphylococcus aureus* heterogeneously resistant to vancomycin.. Lancet.

[7] Itoh J, Omoto S, Shomura T, Nishizawa N, Miyado S (1981). Amicoumacin-A, a new antibiotic with strong antiinflammatory and antiulcer activity.. J Antibiot (Tokyo).

[8] Shimojima Y, Hayashi H, Ooka T, Shibukawa M (1982). Production, isolation and pharmacological studies of AI-77s.. Agric Biol Chem.

[9] Shimojima Y, Hayashi H, Ooka T, Shibukawa M (1982). Studies on AI-77s, microbial products with pharmacological activity - Structures and the chemical nature of AI-77s.. Tet Lett.

[10] Shimojima Y, Hayashi H, Ooka T, Shibukawa M (1984). Studies on AI-77s, microbial products with gastroprotective activity -Structures and the chemical nature of AI-77s.. Tetrahedron.

[11] Azumi M, Ogawa K, Fujita T, Takeshita M, Yoshida R (2008). Bacilosarcins A and B, novel bioactive isocoumarins with unusual heterocyclic cores from the marine-derived bacterium *Bacillus subtlis*.. Tetrahedron.

[12] Canedo LM, Fernandez Puentes JL, Perez Baz J, Acebal C, de la Calle F (1997). PM-94128, a new isocoumarin antitumor agent produced by a marine bacterium.. J Antibiot.

[13] Huang YF, Li LH, Tian L, Qiao L, Hua HM (2006). Sg17-1-4, a novel isocoumarin from a marine fungus *Alternaria tenuis* Sg17-1.. J Antibiot.

[14] McInerney BV, Taylor WC, Lacey MJ, Akhurst RJ, Gregson RP (1991). Biologically active metabolites from *Xenorhabdus* spp., Part 2. Benzopyran-1-one derivatives with gastroprotective activity.. J Nat Prod.

[15] Sato T, Nagai K, Suzuki K, Morioka M, Saito T (1992). A new isocoumarin antibiotic, Y-05460M-A.. J Antibiot.

[16] Pinchuk IV, Bressollier P, Verneuil B, Fenet B, Sorokulova IB (2001). In vitro anti-*Helicobacter pylori* activity of the probiotic strain *Bacillus subtilis* 3 is due to secretion of antibiotics.. Antimicrob Agents Chemother.

[17] Shinabarger DL, Marotti KR, Murray RW, Lin AH, Melchior EP (1997). Mechanism of action of oxazolidinones: effects of linezolid and eperezolid on translation reactions.. Antimicrob Agents Chemother.

[18] Silverman JA, Perlmutter NG, Shapiro HM (2003). Correlation of daptomycin bactericidal activity and membrane depolarization in *Staphylococcus aureus*.. Antimicrob Agents Chemother.

[19] Hashimoto M, Taguchi T, Nishida S, Ueno K, Koizumi K (2007). Isolation of 8′-phosphate ester derivatives of amicoumacins: structure-activity relationship of hydroxy amino acid moiety.. J Antibiot (Tokyo).

[20] Gioia J, Yerrapragada S, Qin X, Jiang H, Igboeli OC (2007). Paradoxical DNA repair and peroxide resistance gene conservation in *Bacillus pumilus* SAFR-032.. PLoS One.

[21] Nakano MM, Corbell N, Besson J, Zuber P (1992). Isolation and characterization of *sfp* - a gene that functions in the production of the lipopeptide biosurfactant, surfactin, in *Bacillus subtilis*.. Mol Gen Genet.

[22] Lambalot RH, Gehring AM, Flugel RS, Zuber P, LaCelle M (1996). A new enzyme superfamily - The phosphopantetheinyl transferases.. Chem Biol.

[23] Park D, Ciezki K, van der Hoeven R, Singh S, Reimer D (2009). Genetic analysis of xenocoumacin antibiotic production in the mutualistic bacterium *Xenorhabdus nematophila*.. Mol Microbiol.

[24] Brazas MD, Hancock RE (2005). Using microarray gene signatures to elucidate mechanisms of antibiotic action and resistance.. Drug Discov Today.

[25] Kenny JG, Ward D, Josefsson E, Jonsson IM, Hinds J (2009). The *Staphylococcus aureus* response to unsaturated long chain free fatty acids: survival mechanisms and virulence implications.. PLoS One.

[26] Bischoff M, Dunman P, Kormanec J, Macapagal D, Murphy E (2004). Microarray-based analysis of the *Staphylococcus aureus* sigmaB regulon.. J Bacteriol.

[27] Pane-Farre J, Jonas B, Forstner K, Engelmann S, Hecker M (2006). The sigmaB regulon in *Staphylococcus aureus* and its regulation.. Int J Med Microbiol.

[28] Hecker M, Pane-Farre J, Volker U (2007). SigB-dependent general stress response in *Bacillus subtilis* and related gram-positive bacteria.. Annu Rev Microbiol.

[29] Price CW, Sonenshein AL, Hoch JA, Losick R (2002). General stress response.. *Bacillus subtilis* and its closest relatives.

[30] Groicher KH, Firek BA, Fujimoto DF, Bayles KW (2000). The *Staphylococcus aureus lrgAB* operon modulates murein hydrolase activity and penicillin tolerance.. J Bacteriol.

[31] Rice KC, Firek BA, Nelson JB, Yang SJ, Patton TG (2003). The *Staphylococcus aureus cidAB* operon: evaluation of its role in regulation of murein hydrolase activity and penicillin tolerance.. J Bacteriol.

[32] Stapleton MR, Horsburgh MJ, Hayhurst EJ, Wright L, Jonsson IM (2007). Characterization of IsaA and SceD, two putative lytic transglycosylases of *Staphylococcus aureus*.. J Bacteriol.

[33] Ramadurai L, Lockwood KJ, Nadakavukaren MJ, Jayaswal RK (1999). Characterization of a chromosomally encoded glycylglycine endopeptidase of *Staphylococcus aureus*.. Microbiology.

[34] Oshida T, Sugai M, Komatsuzawa H, Hong YM, Suginaka H (1995). A *Staphylococcus aureus* autolysin that has an N-acetylmuramoyl-L-alanine amidase domain and an endo-beta-N-acetylglucosaminidase domain: cloning, sequence analysis, and characterization.. Proc Natl Acad Sci USA.

[35] Kuroda M, Ohta T, Hayashi H (1995). Isolation and the gene cloning of an alkaline shock protein in methicillin resistant *Staphylococcus aureus*.. Biochem Biophys Res Commun.

[36] Bayles KW (2007). The biological role of death and lysis in biofilm development.. Nat Rev Microbiol.

[37] Heilmann C, Hartleib J, Hussain MS, Peters G (2005). The multifunctional *Staphylococcus aureus* autolysin aaa mediates adherence to immobilized fibrinogen and fibronectin.. Infect Immun.

[38] Baba T, Schneewind O (1998). Targeting of muralytic enzymes to the cell division site of Gram-positive bacteria: repeat domains direct autolysin to the equatorial surface ring of *Staphylococcus aureus*.. EMBO J.

[39] Schlag M, Biswas R, Krismer B, Kohler T, Zoll S (2010). Role of staphylococcal wall teichoic acid in targeting the major autolysin Atl.. Mol Microbiol.

[40] Mwangi MM, Wu SW, Zhou Y, Sieradzki K, de Lencastre H (2007). Tracking the in vivo evolution of multidrug resistance in *Staphylococcus aureus* by whole-genome sequencing.. Proc Natl Acad Sci USA.

[41] Johanson U, Hughes D (1994). Fusidic acid-resistant mutants define three regions in elongation factor G of *Salmonella typhimurium*.. Gene.

[42] Chen HJ, Hung WC, Tseng SP, Tsai JC, Hsueh PR (2010). Fusidic acid resistance determinants in *Staphylococcus aureus* clinical isolates.. Antimicrob Agents Chemother.

[43] Norstrom T, Lannergard J, Hughes D (2007). Genetic and phenotypic identification of fusidic acid-resistant mutants with the small-colony-variant phenotype in *Staphylococcus aureus*.. Antimicrob Agents Chemother.

[44] Helser TL, Davies JE, Dahlberg JE (1971). Change in methylation of 16 S ribosomal RNA associated with mutation to kasugamycin resistance in *Escherichia coli*.. Nat New Biol.

[45] AEvarsson A, Brazhnikov E, Garber M, Zheltonosova J, Chirgadze Y (1994). Three-dimensional structure of the ribosomal translocase: elongation factor G from *Thermus thermophilus*.. EMBO J.

[46] Laurberg M, Kristensen O, Martemyanov K, Gudkov AT, Nagaev I (2000). Structure of a mutant EF-G reveals domain III and possibly the fusidic acid binding site.. J Mol Biol.

[47] Patton TG, Yang SJ, Bayles KW (2006). The role of proton motive force in expression of the *Staphylococcus aureus cid* and *lrg* operons.. Mol Microbiol.

[48] Muthaiyan A, Silverman JA, Jayaswal RK, Wilkinson BJ (2008). Transcriptional profiling reveals that daptomycin induces the *Staphylococcus aureus* cell wall stress stimulon and genes responsive to membrane depolarization.. Antimicrob Agents Chemother.

[49] Wang D, Jin Q, Xiang H, Wang W, Guo N (2011). Transcriptional and functional analysis of the effects of magnolol: inhibition of autolysis and biofilms in *Staphylococcus aureus*.. PLoS One.

[50] Renzoni A, Barras C, Francois P, Charbonnier Y, Huggler E (2006). Transcriptomic and functional analysis of an autolysis-deficient, teicoplanin-resistant derivative of methicillin-resistant *Staphylococcus aureus*.. Antimicrob Agents Chemother.

[51] Takahashi J, Komatsuzawa H, Yamada S, Nishida T, Labischinski H (2002). Molecular characterization of an atl null mutant of *Staphylococcus aureus*.. Microbiol Immunol.

[52] Houston P, Rowe SE, Pozzi C, Waters EM, O'Gara JP (2010). Essential role for the major autolysin in the fibronectin-binding protein-mediated *Staphylococcus aureus* biofilm phenotype.. Infect Immun.

[53] Sugai M, Yamada S, Nakashima S, Komatsuzawa H, Matsumoto A (1997). Localized perforation of the cell wall by a major autolysin: atl gene products and the onset of penicillin-induced lysis of *Staphylococcus aureus*.. J Bacteriol.

[54] Igo MM, Losick R (1986). Regulation of a promoter that is utilized by minor forms of RNA polymerase holoenzyme in *Bacillus subtilis*.. J Mol Biol.

[55] Charbonnier Y, Gettler B, Francois P, Bento M, Renzoni A (2005). A generic approach for the design of whole-genome oligoarrays, validated for genomotyping, deletion mapping and gene expression analysis on *Staphylococcus aureus*.. BMC Genomics.

[56] Becher D, Hempel K, Sievers S, Zuhlke D, Pane-Farre J (2009). A proteomic view of an important human pathogen–towards the quantification of the entire *Staphylococcus aureus* proteome.. PLoS One.

[57] Saeed AI, Bhagabati NK, Braisted JC, Liang W, Sharov V (2006). TM4 microarray software suite.. Methods Enzymol.

[58] Gertz S, Engelmann S, Schmid R, Ohlsen K, Hacker J (1999). Regulation of sigmaB-dependent transcription of *sigB* and *asp23* in two different *Staphylococcus aureus* strains.. Mol Gen Genet.

[59] Wetzstein M, Volker U, Dedio J, Lobau S, Zuber U (1992). Cloning, sequencing, and molecular analysis of the dnaK locus from *Bacillus subtilis*.. J Bacteriol.

[60] de Jonge BL, Chang YS, Gage D, Tomasz A (1992). Peptidoglycan composition of a highly methicillin-resistant *Staphylococcus aureus* strain. The role of penicillin binding protein 2A.. J Biol Chem.

[61] Arnaud M, Chastanet A, Debarbouille M (2004). New vector for efficient allelic replacement in naturally nontransformable, low-GC-content, gram-positive bacteria.. Appl Environ Microbiol.

[62] Stragier P, Bonamy C, Karmazyn-Campelli C (1988). Processing of a sporulation factor in *Bacillus subtilis*: How morphological structure could control gene expression.. Cell.

