# Supplementary material for: Response of Methicillin-Resistant Staphylococcus aureus to Amicoumacin A
Source: PLoS One. 2012 Mar 30;7(3):e34037. doi: 10.1371/journal.pone.0034037 (PMC3316591; doi:10.1371/journal.pone.0034037)

S. aureus COL Locus	Gene	t0 v t10[1]	t0 v t40[1]	t10 v t40[1]	expr.[2]	SigB[3]	linoleic acid[4]	functional gene annotation	cellular role category
SACOL0247	lrgA	8.64	-1.04	-8.99	C1		↓	holin-like protein LrgA	Cell envelope: Biosynthesis and degradation of murein sacculus and peptidoglycan
SACOL0248	lrgB	14.03	1.45	-9.69	C1			lrgB protein	Cell envelope: Biosynthesis and degradation of murein sacculus and peptidoglycan
SACOL0740	-	2.65	-1.24	-3.30	C1	σ^B	↑	decarboxylase family protein	Unknown function: Enzymes of unknown specificity
SACOL0741	-	3.18	1.30	-2.45	C1	σ^B	↑	conserved hypothetical protein	Hypothetical proteins: Conserved
SACOL0742	-	3.43	1.29	-2.66	C1	σ^B		conserved hypothetical protein	Hypothetical proteins: Conserved
SACOL0866	-	5.43	1.98	-2.74	C1			hypothetical protein	-
SACOL1074	purK	2.31	-1.34	-3.09	C1			phosphoribosylaminoimidazole carboxylase, ATPase subunit	Purines, pyrimidines, nucleosides, and nucleotides: Purine ribonucleotide biosynthesis
SACOL1076	purS	2.22	-1.13	-2.50	C1			phosphoribosylformylglycinamidine synthase, PurS protein	Purines, pyrimidines, nucleosides, and nucleotides: Purine ribonucleotide biosynthesis
SACOL1077	purQ	2.54	-1.86	-4.71	C1			phosphoribosylformylglycinamidine synthase I	Purines, pyrimidines, nucleosides, and nucleotides: Purine ribonucleotide biosynthesis
SACOL1078	purL	2.10	-1.62	-3.41	C1			phosphoribosylformylglycinamidine synthase II	Purines, pyrimidines, nucleosides, and nucleotides: Purine ribonucleotide biosynthesis
SACOL1080	purM	3.11	1.47	-2.12	C1			phosphoribosylformylglycinamidine cyclo-ligase	Purines, pyrimidines, nucleosides, and nucleotides: Purine ribonucleotide biosynthesis
SACOL1090	-	2.35	-1.47	-3.45	C1	σ^B	↑	conserved hypothetical protein	Hypothetical proteins: Conserved
SACOL1226	-	2.86	-1.06	-3.03	C1			conserved hypothetical protein	Hypothetical proteins: Conserved
SACOL1236	-	1.79	-1.54	-2.76	C1			thiamine pyrophosphokinase	Biosynthesis of cofactors, prosthetic groups, and carriers: Thiamine
SACOL1373	-	3.75	1.47	-2.55	C1			conserved hypothetical protein	Hypothetical proteins: Conserved
SACOL1398	-	1.89	-1.41	-2.67	C1			transcriptional regulator, putative	Regulatory functions: Other
SACOL1602	-	2.25	-1.61	-3.63	C1		↑	metallo-beta-lactamase family protein	Unknown function: Enzymes of unknown specificity
SACOL1647	-	1.96	-1.46	-2.86	C1			conserved hypothetical protein	Hypothetical proteins: Conserved
SACOL1735	coaE	2.09	-1.41	-2.95	C1			dephospho-CoA kinase	Biosynthesis of cofactors, prosthetic groups, and carriers: Pantothenate and coenzyme A
SACOL1745	pyk	2.67	-1.66	-4.42	C1			pyruvate kinase	Energy metabolism: Glycolysis/gluconeogenesis
SACOL1789	-	2.23	-1.34	-3.00	C1	σ^B		conserved hypothetical protein	Hypothetical proteins: Conserved
SACOL1895	-	3.11	1.17	-2.66	C1			conserved hypothetical protein	Hypothetical proteins: Conserved
SACOL1897	-	2.39	-1.06	-2.55	C1			protein export protein PrsA, putative	Protein fate: Protein and peptide secretion and trafficking
SACOL1898	cbf1	2.51	1.19	-2.11	C1			cmp-binding-factor 1	Mobile and extrachromosomal element functions: Plasmid functions
SACOL1912	-	2.65	1.45	-1.82	C1	σ^B		glucosamine-6-phosphate isomerase, putative	Central intermediary metabolism: Amino sugars
SACOL1933	-	4.68	1.76	-2.66	C1	σ^B	↑	ThiJ/PfpI family protein	Unknown function: General
SACOL1934	-	3.37	1.50	-2.24	C1			conserved hypothetical protein	Hypothetical proteins: Conserved
SACOL1941	yihY	2.95	-1.13	-3.34	C1	σ^B		YihY family protein	Unknown function: General
SACOL2091	fabZ	4.02	-1.02	-4.08	C1	σ^B	↑	beta-hydroxyacyl-(acyl-carrier-protein) dehydratase FabZ	Fatty acid and phospholipid metabolism: Biosynthesis
SACOL2132	-	2.50	1.46	-1.71	C1	σ^B		conserved hypothetical protein	Hypothetical proteins: Conserved
SACOL2136	-	4.11	1.38	-2.97	C1	σ^B	↑	conserved hypothetical protein	Hypothetical proteins: Conserved
SACOL2174	-	5.38	1.74	-3.09	C1	σ^B	↑	conserved hypothetical protein	Hypothetical proteins: Conserved
SACOL2175	-	5.70	1.29	-4.41	C1	σ^B	↑	conserved hypothetical protein	Hypothetical proteins: Conserved
SACOL2242	-	2.32	-1.10	-2.55	C1			xanthine/uracil permease family protein	Transport and binding proteins: Nucleosides, purines and pyrimidines
SACOL2300	-	3.13	1.09	-2.87	C1	σ^B	↑	conserved hypothetical protein	Hypothetical proteins: Conserved
SACOL2301	-	3.45	1.47	-2.35	C1	σ^B	↑	formate dehydrogenase, alpha subunit, putative	Energy metabolism: Anaerobic
SACOL2302	-	2.51	-1.19	-2.98	C1		↑	transcriptional regulator, putative	Regulatory functions: Other
SACOL2321	-	3.42	1.58	-2.16	C1	σ^B	↑	oxidoreductase, short chain dehydrogenase/reductase family	Unknown function: Enzymes of unknown specificity
SACOL2327	hutG	2.57	-1.06	-2.74	C1	σ^B	↑	formiminoglutamase	Energy metabolism: Amino acids and amines
SACOL2329	rpiA	2.76	-1.40	-3.86	C1			ribose 5-phosphate isomerase	Energy metabolism: Pentose phosphate pathway
SACOL2379	-	3.36	1.32	-2.55	C1	σ^B	↑	conserved hypothetical protein	Hypothetical proteins: Conserved
SACOL2394	narH	3.26	1.20	-2.73	C1		↑	respiratory nitrate reductase, beta subunit	Energy metabolism: Anaerobic; Energy metabolism: Electron transport; Central intermediary metabolism: Nitrogen metabolism
SACOL2395	narG	3.49	1.17	-2.98	C1			respiratory nitrate reductase, alpha subunit	Energy metabolism: Anaerobic; Energy metabolism: Electron transport; Central intermediary metabolism: Nitrogen metabolism
SACOL2563	clpL	2.53	1.08	-2.34	C1	σ^B	↑	ATP-dependent Clp protease, putative	Protein fate: Degradation of proteins, peptides, and glycopeptides
SACOL2578	-	1.88	-1.37	-2.59	C1	σ^B	↑	glycosyl transferase, group 2 family protein	Cell envelope: Biosynthesis and degradation of surface polysaccharides and lipopolysaccharides
SACOL2596	-	2.89	1.14	-2.53	C1	σ^B	↑	conserved hypothetical protein	Hypothetical proteins: Conserved
SACOL2597	-	3.50	1.39	-2.52	C1	σ^B	↑	hydrolase, alpha/beta hydrolase fold family	Unknown function: Enzymes of unknown specificity
SACOL2609	-	2.31	-1.79	-4.14	C1			conserved hypothetical protein	Hypothetical proteins: Conserved
SACOL2621	-	2.94	1.03	-2.86	C1			hypothetical protein	-
SACOL2625	-	3.33	1.18	-2.83	C1	σ^B	↑	conserved hypothetical protein	Hypothetical proteins: Conserved
SACOL2681	-	2.90	1.54	-1.88	C1	σ^B	↑	conserved hypothetical protein	Hypothetical proteins: Conserved
SACOL2717	-	2.83	-1.21	-3.42	C1			hypothetical protein	-
SACOL0012	-	1.06	2.84	2.69	C2			homoserine O-acetyltransferase, putative	Amino acid biosynthesis: Aspartate family
SACOL0184	-	1.25	4.03	3.23	C2			peptide ABC transporter, ATP-binding protein	Transport and binding proteins: Amino acids, peptides and amines
SACOL0263	lytM	1.12	3.86	3.43	C2			peptidoglycan hydrolase	Cell envelope: Biosynthesis and degradation of murein sacculus and peptidoglycan
SACOL0428	metE	1.18	2.80	2.38	C2			5-methyltetrahydropteroyltriglutamate--homocysteine methyltransferase	Amino acid biosynthesis: Aspartate family
SACOL0504	-	-1.19	4.44	5.29	C2			ABC transporter, ATP-binding protein	Transport and binding proteins: Unknown substrate
SACOL0505	-	1.33	6.67	5.00	C2			ABC transporter, permease protein	Transport and binding proteins: Unknown substrate
SACOL0514	gltB	-1.03	3.48	3.57	C2			glutamate synthase, large subunit	Amino acid biosynthesis: Glutamate family
SACOL0515	gltD	1.21	3.87	3.19	C2			glutamate synthase, small subunit	Amino acid biosynthesis: Glutamate family
SACOL0517	treC	1.18	3.23	2.74	C2			alpha,alpha-phosphotrehalase	Energy metabolism: Biosynthesis and degradation of polysaccharides
SACOL0641	-	1.11	2.62	2.37	C2		↑	conserved hypothetical protein	Hypothetical proteins: Conserved
SACOL0712	-	-1.16	3.11	3.60	C2			lipase/esterase	Fatty acid and phospholipid metabolism: Degradation
SACOL0770	-	1.21	4.81	3.96	C2			radical activating enzyme family protein	Unknown function: Enzymes of unknown specificity
SACOL0771	-	1.30	5.31	4.09	C2		↑	6-pyruvoyl tetrahydrobiopterin synthase, putative	Biosynthesis of cofactors, prosthetic groups, and carriers: Other
SACOL0772	-	1.01	4.29	4.25	C2			exsB protein	Unknown function: General
SACOL0802	-	-1.17	3.77	4.43	C2		↓	conserved hypothetical protein	Hypothetical proteins: Conserved
SACOL0813	-	1.26	3.07	2.43	C2			comf operon protein 1, putative	Cellular processes: DNA transformation
SACOL0845	est	1.26	3.31	2.63	C2			carboxylesterase	Cellular processes: Detoxification; Fatty acid and phospholipid metabolism: Degradation
SACOL0870	-	1.20	2.66	2.22	C2			LysE/YggA family protein	Unknown function: General
SACOL0882	-	-1.21	4.53	5.49	C2			ABC transporter, ATP-binding protein	Transport and binding proteins: Unknown substrate
SACOL0883	-	-1.06	5.19	5.50	C2			ABC transporter, permease protein	Transport and binding proteins: Unknown substrate
SACOL0884	-	1.10	4.39	3.98	C2			ABC transporter, substrate-binding protein	Transport and binding proteins: Unknown substrate
SACOL1032	-	-1.15	3.87	4.44	C2			ComK family protein	Unknown function: General
SACOL1268	gid	1.09	2.60	2.39	C2		↑	Gid protein	Unknown function: General
SACOL1375	-	1.18	3.67	3.11	C2			conserved hypothetical protein	Hypothetical proteins: Conserved
SACOL1414	-	1.19	3.08	2.59	C2			peptide ABC transporter, ATP-binding protein	Transport and binding proteins: Amino acids, peptides and amines
SACOL1415	-	1.09	3.06	2.80	C2			peptide ABC transporter, ATP-binding protein	Transport and binding proteins: Amino acids, peptides and amines
SACOL1416	-	1.00	4.13	4.12	C2			peptide ABC transporter, permease protein, putative	Transport and binding proteins: Amino acids, peptides and amines
SACOL1417	-	1.26	5.78	4.60	C2			peptide ABC transporter, permease protein	Transport and binding proteins: Amino acids, peptides and amines
SACOL1442	-	1.14	2.66	2.33	C2			IS1272-related, transposase, degenerate	Mobile and extrachromosomal element functions: Transposon functions; Disrupted reading frame
SACOL1468	-	1.24	2.89	2.33	C2			membrane protein, putative	Cell envelope: Other
SACOL1490	pbp2	1.17	2.59	2.21	C2			penicillin-binding protein 2	Cell envelope: Biosynthesis and degradation of murein sacculus and peptidoglycan
SACOL1541	-	1.34	4.01	3.00	C2		↓	transcriptional regulator, Fur family	Transport and binding proteins: Cations and iron carrying compounds; Regulatory functions: DNA interactions
SACOL1724	-	1.14	3.14	2.75	C2			MutT/nudix family protein	DNA metabolism: DNA replication, recombination, and repair

SACOL1830	-	-1.13	2.41	2.73	C2			abortive infection protein family	Unknown function: General
SACOL1870	-	1.02	3.18	3.12	C2			hypothetical protein	-
SACOL1880	lukD	1.15	2.78	2.42	C2			leukotoxin LukD	Cellular processes: Toxin production and resistance; Cellular processes: Pathogenesis
SACOL1881	lukE	-1.17	2.82	3.29	C2			leukotoxin LukE	Cellular processes: Toxin production and resistance; Cellular processes: Pathogenesis
SACOL1954	-	1.07	3.24	3.04	C2			exonuclease	Unknown function: Enzymes of unknown specificity
SACOL2020	-	-1.05	3.47	3.66	C2			conserved hypothetical protein	Hypothetical proteins: Conserved
SACOL2033	-	-1.03	4.20	4.32	C2			conserved hypothetical protein	Hypothetical proteins: Conserved
SACOL2034	-	-1.01	4.54	4.60	C2			conserved hypothetical protein	Hypothetical proteins: Conserved
SACOL2066	kdpC	1.24	3.59	2.89	C2			K+-transporting ATPase, C subunit	Transport and binding proteins: Cations and iron carrying compounds
SACOL2081	-	-1.03	4.59	4.75	C2			hypothetical protein	-
SACOL2322	-	1.28	3.49	2.72	C2			peptidase, M20/M25/M40 family	Protein fate: Degradation of proteins, peptides, and glycopeptides
SACOL2458	-	1.06	4.25	3.99	C2			amino acid permease	Transport and binding proteins: Amino acids, peptides and amines
SACOL2466	-	1.16	5.43	4.69	C2			hypothetical protein	-
SACOL2483	-	1.26	3.07	2.43	C2			transporter, putative	Transport and binding proteins: Unknown substrate
SACOL2544	sdaAA	1.11	4.00	3.62	C2			L-serine dehydratase, iron-sulfur-dependent, alpha subunit	Energy metabolism: Amino acids and amines
SACOL2545	sdaAB	1.07	6.96	6.53	C2			L-serine dehydratase, iron-sulfur-dependent, beta subunit	Energy metabolism: Amino acids and amines
SACOL2546	-	1.06	6.29	5.91	C2			perfringolysin O regulator protein, putative	Regulatory functions: Other
SACOL2588	-	1.03	2.90	2.82	C2			hypothetical protein	-
SACOL2615	panB	1.03	2.97	2.88	C2			3-methyl-2-oxobutanoate hydroxymethyltransferase	Biosynthesis of cofactors, prosthetic groups, and carriers: Pantothenate and coenzyme A
SACOL0007	-	1.55	2.74	1.76	C3			YjeF-related protein	Unknown function: General
SACOL0061	-	1.55	2.61	1.68	C3			conserved domain protein	Hypothetical proteins: Domain
SACOL0129	-	1.36	2.79	2.05	C3			conserved hypothetical protein	Hypothetical proteins: Conserved
SACOL0178	-	1.78	2.74	1.54	C3			PTS system, IIBC components	Transport and binding proteins: Carbohydrates, organic alcohols, and acids; Signal transduction: PTS
SACOL0210	-	1.57	3.08	1.97	C3			hypothetical protein	-
SACOL0212	-	1.61	3.13	1.95	C3			3-hydroxyacyl-CoA dehydrogenase protein	Fatty acid and phospholipid metabolism: Degradation
SACOL0231	-	1.62	3.27	2.02	C3			hypothetical protein	-
SACOL0308	-	1.44	2.71	1.89	C3			carbohydrate kinase, PfkB family	Energy metabolism: Sugars
SACOL0309	-	1.64	3.82	2.33	C3			conserved hypothetical protein	Hypothetical proteins: Conserved
SACOL0310	-	2.29	4.54	1.99	C3			nucleoside permease NupC, putative	Transport and binding proteins: Nucleosides, purines and pyrimidines
SACOL0324	-	1.51	2.74	1.81	C3			hypothetical protein	-
SACOL0333	-	1.60	2.50	1.56	C3			conserved hypothetical protein	Hypothetical proteins: Conserved
SACOL0334	-	1.57	2.56	1.63	C3			conserved hypothetical protein	Hypothetical proteins: Conserved
SACOL0413	-	1.57	2.65	1.68	C3			ribosomal-protein-serine acetyltransferase, putative	Protein synthesis: Ribosomal proteins: synthesis and modification
SACOL0414	-	1.57	3.53	2.25	C3			lipoprotein, putative	Cell envelope: Other
SACOL0415	-	1.31	2.75	2.10	C3			Dyp-type peroxidase family protein	Unknown function: Enzymes of unknown specificity
SACOL0442	-	1.64	2.59	1.58	C3			staphylococcal enterotoxin, putative	Cellular processes: Toxin production and resistance
SACOL0462	-	1.47	2.62	1.78	C3			hypothetical protein	-
SACOL0754	norA	1.64	3.62	2.20	C3			multi drug resistance protein (norA)	Cellular processes: Toxin production and resistance; Transport and binding proteins: Other
SACOL0757	-	1.59	3.79	2.38	C3			transcriptional regulator, DeoR family	Regulatory functions: DNA interactions
SACOL0758	fruK	1.69	4.58	2.70	C3		↓	1-phosphofructokinase	Energy metabolism: Glycolysis/gluconeogenesis
SACOL0804	-	2.05	3.45	1.69	C3			conserved hypothetical protein	Hypothetical proteins: Conserved
SACOL0814	-	1.43	2.95	2.06	C3			competence protein F	Cellular processes: DNA transformation
SACOL0820	-	1.31	2.68	2.05	C3			LysM domain protein	Unknown function: General
SACOL0846	-	2.07	4.05	1.96	C3		↑	exoribonuclease, VacB/RNase II family	Transcription: Degradation of RNA
SACOL0847	smpB	2.09	4.17	2.00	C3		↑	SsrA-binding protein	Protein synthesis: Other
SACOL0855	-	1.96	4.32	2.20	C3		↑	acetyltransferase, GNAT family	Unknown function: Enzymes of unknown specificity
SACOL0865	-	1.68	2.79	1.66	C3			hypothetical protein	-
SACOL0998	-	1.51	2.89	1.91	C3			oligopeptide ABC transporter, ATP-binding protein	Transport and binding proteins: Amino acids, peptides and amines
SACOL1107	-	1.81	3.22	1.78	C3			transcriptional regulator, Cro/CI family	Regulatory functions: DNA interactions
SACOL1182	arcC1	1.74	2.62	1.51	C3		↓	carbamate kinase	Energy metabolism: Amino acids and amines
SACOL1238	rpmB	1.66	2.73	1.64	C3			ribosomal protein L28	Protein synthesis: Ribosomal proteins: synthesis and modification
SACOL1362	hom	1.40	4.65	3.32	C3			homoserine dehydrogenase	Amino acid biosynthesis: Aspartate family
SACOL1363	thrC	1.33	3.44	2.59	C3			threonine synthase	Amino acid biosynthesis: Aspartate family
SACOL1400	-	1.32	2.94	2.22	C3			ImpB/MucB/SamB family protein	DNA metabolism: DNA replication, recombination, and repair
SACOL1488	-	1.35	2.88	2.13	C3			hypothetical protein	-
SACOL1489	recU	1.57	3.60	2.29	C3			recombination protein U	DNA metabolism: DNA replication, recombination, and repair
SACOL1525	fer	1.54	4.66	3.03	C3			ferredoxin	Energy metabolism: Electron transport
SACOL1540	xerD	1.39	4.28	3.08	C3			tyrosine recombinase XerD	DNA metabolism: DNA replication, recombination, and repair
SACOL1696	ruvB	1.52	2.55	1.68	C3			Holliday junction DNA helicase RuvB	DNA metabolism: DNA replication, recombination, and repair
SACOL1698	-	1.96	3.08	1.57	C3			ACT domain protein	Unknown function: General
SACOL1699	-	1.83	2.82	1.54	C3			GTP-binding protein, GTP1/OBG family	Unknown function: General
SACOL1702	rplU	1.70	2.76	1.62	C3			ribosomal protein L21	Protein synthesis: Ribosomal proteins: synthesis and modification
SACOL1723	-	1.54	3.28	2.13	C3			conserved hypothetical protein	Hypothetical proteins: Conserved
SACOL1822	arsR	1.45	4.93	3.41	C3			arsenical resistance operon repressor	Cellular processes: Detoxification; Regulatory functions: DNA interactions
SACOL1823	arsB	1.70	4.60	2.71	C3			arsenical pump membrane protein	Cellular processes: Detoxification; Transport and binding proteins: Other
SACOL1824	arsC	1.51	3.27	2.17	C3			arsenate reductase (thioredoxin)	Cellular processes: Detoxification
SACOL1825	-	1.58	2.99	1.89	C3			N-acetylmuramoyl-L-alanine amidase, family 4	Cell envelope: Biosynthesis and degradation of surface polysaccharides and lipopolysaccharides
SACOL2088	sceD	2.38	11.30	4.75	C3	σ^B		sceD protein, putative	Unknown function: General
SACOL2215	rpsM	1.37	2.73	1.99	C3		↑	ribosomal protein S13/S18	Protein synthesis: Ribosomal proteins: synthesis and modification
SACOL2256	-	1.77	2.78	1.58	C3			transcriptional regulator, MarR family	Cellular processes: Toxin production and resistance; Regulatory functions: DNA interactions
SACOL2291	-	1.59	3.57	2.24	C3	σ^B		staphyloxanthin biosynthesis protein	Cellular processes: Pathogenesis
SACOL2353	tcaR	1.95	5.71	2.93	C3		↑	transcriptional regulator TcaR	Cell envelope: Biosynthesis and degradation of murein sacculus and peptidoglycan; Toxin production and resistance; Regulatory functions: DNA interactions
SACOL2416	-	1.28	2.78	2.17	C3			cation efflux family protein	Transport and binding proteins: Cations and iron carrying compounds
SACOL2441	-	1.39	3.41	2.44	C3			amino acid permease	Transport and binding proteins: Amino acids, peptides and amines
SACOL2464	-	2.34	5.64	2.41	C3			addiction module toxin, Txe/YoeB family	Cellular processes: Toxin production and resistance; Mobile and extrachromosomal element functions: Other
SACOL2465	-	2.28	4.96	2.17	C3			addiction module antitoxin, Axe family	Cellular processes: Toxin production and resistance; Mobile and extrachromosomal element functions: Other
SACOL2614	panC	1.39	3.09	2.22	C3			pantoate--beta-alanine ligase	Biosynthesis of cofactors, prosthetic groups, and carriers: Pantothenate and coenzyme A
SACOL2632	cudT	1.32	2.85	2.15	C3			osmoprotectant transporter, BCCT family	Transport and binding proteins: Amino acids, peptides and amines
SACOL2710	-	1.37	2.72	1.99	C3			conserved hypothetical protein	Hypothetical proteins: Conserved
SACOL2713	-	2.39	4.93	2.06	C3			rhodanese-like domain protein	Unknown function: General
SACOL2714	pcp	1.33	3.61	2.72	C3			pyrrolidone-carboxylate peptidase	Protein fate: Degradation of proteins, peptides, and glycopeptides
SACOL0008	hutH	3.87	4.16	1.08	C4			histidine ammonia-lyase	Energy metabolism: Amino acids and amines
SACOL0065	-	3.41	2.83	-1.20	C4			conserved hypothetical protein	Hypothetical proteins: Conserved
SACOL0073	-	2.41	2.59	1.08	C4			conserved hypothetical protein	Hypothetical proteins: Conserved
SACOL0122	-	2.10	2.61	1.24	C4		↑	tetracycline resistance protein, putative	Cellular processes: Toxin production and resistance; Transport and binding proteins: Other
SACOL0136	cap5A	3.80	2.67	-1.43	C4	σ^B		capsular polysaccharide biosynthesis protein Cap5A	Cell envelope: Biosynthesis and degradation of surface polysaccharides and lipopolysaccharides; Transport and binding proteins: Carbohydrates, organic alcohols, and acids

SACOL0137	cap5B	3.01	2.30	-1.31	C4	σ^B		capsular polysaccharide biosynthesis protein Cap5B	Cell envelope: Biosynthesis and degradation of surface polysaccharides and lipopolysaccharides
SACOL0138	cap5C	3.37	1.99	-1.69	C4	σ^B		capsular polysaccharide biosynthesis protein Cap5C	Cell envelope: Biosynthesis and degradation of surface polysaccharides and lipopolysaccharides
SACOL0264	-	3.93	4.44	1.13	C4			ABC transporter, ATP-binding protein	Transport and binding proteins: Unknown substrate
SACOL0265	-	3.13	4.97	1.59	C4			hypothetical protein	-
SACOL0317	-	2.37	3.79	1.60	C4			lipase precursor, interruption-N	Fatty acid and phospholipid metabolism: Degradation; Disrupted reading frame
SACOL0342	-	1.95	2.73	1.40	C4			hypothetical protein	-
SACOL0343	-	1.98	2.61	1.32	C4			prophage L54a, replicative DNA helicase, putative	DNA metabolism: DNA replication, recombination, and repair; Mobile and extrachromosomal element functions: Prophage functions
SACOL0399	-	4.30	1.99	-2.16	C4	σ^B	↑	oxidoreductase, putative	Unknown function: Enzymes of unknown specificity
SACOL0437	rpsF	2.12	2.67	1.26	C4			ribosomal protein S6	Protein synthesis: Ribosomal proteins: synthesis and modification
SACOL0438	ssb2	2.05	2.75	1.34	C4		↑	single-stranded DNA-binding protein	DNA metabolism: DNA replication, recombination, and repair
SACOL0446	-	2.54	1.79	-1.42	C4	σ^B	↑	conserved hypothetical protein	Hypothetical proteins: Conserved
SACOL0447	-	2.59	2.65	1.02	C4			phosphoglycerate mutase family protein	Unknown function: General
SACOL0585	rplJ	4.41	6.46	1.46	C4			ribosomal protein L10	Protein synthesis: Ribosomal proteins: synthesis and modification
SACOL0620	proP	2.74	3.43	1.25	C4			osmoprotectant proline transporter	Transport and binding proteins: Amino acids, peptides and amines
SACOL0630	-	2.91	1.93	-1.51	C4	σ^B	↑	amino acid permease	Transport and binding proteins: Amino acids, peptides and amines
SACOL0671	-	3.86	2.25	-1.72	C4	σ^B	↑	hydrolase, alpha/beta hydrolase fold family	Unknown function: Enzymes of unknown specificity
SACOL0673	-	3.11	4.24	1.36	C4			hypothetical protein	-
SACOL0678	-	4.16	3.04	-1.37	C4	σ^B		integrase/recombinase, phage integrase family	DNA metabolism: DNA replication, recombination, and repair
SACOL0679	-	3.11	2.78	-1.12	C4	σ^B		Na+/H+ antiporter, MnhA component, putative	Transport and binding proteins: Cations and iron carrying compounds
SACOL0680	-	2.95	3.08	1.04	C4	σ^B		Na+/H+ antiporter, MnhB component, putative	Transport and binding proteins: Cations and iron carrying compounds
SACOL0681	-	3.33	3.50	1.05	C4	σ^B		Na+/H+ antiporter, MnhC component, putative	Transport and binding proteins: Cations and iron carrying compounds
SACOL0682	-	3.03	3.13	1.03	C4	σ^B		Na+/H+ antiporter, MnhD component, putative	Transport and binding proteins: Cations and iron carrying compounds
SACOL0684	-	2.62	2.84	1.08	C4	σ^B		Na+/H+ antiporter, MnhE component, putative	Transport and binding proteins: Cations and iron carrying compounds
SACOL0685	-	2.46	2.87	1.17	C4	σ^B		Na+/H+ antiporter, MnhF component, putative	Transport and binding proteins: Cations and iron carrying compounds
SACOL0686	-	2.42	2.62	1.08	C4	σ^B		Na+/H+ antiporter, MnhG component, putative	Transport and binding proteins: Cations and iron carrying compounds
SACOL0756	-	2.70	4.93	1.83	C4			ebsC protein	Unknown function: General
SACOL0768	-	3.39	2.55	-1.33	C4		↓	lipoprotein, putative	Hypothetical proteins: Conserved
SACOL0789	-	3.06	3.09	1.01	C4			GTP cyclohydrolase I family protein	Unknown function: Enzymes of unknown specificity
SACOL0790	-	1.77	2.51	1.41	C4			integral membrane domain protein	Unknown function: General
SACOL0854	-	3.56	5.14	1.44	C4		↑	hypothetical protein	-
SACOL0856	clfA	3.30	3.01	-1.09	C4	σ^B	↑	clumping factor A	Cellular processes: Pathogenesis
SACOL0871	-	3.28	6.21	1.89	C4			acetyltransferase, putative	Unknown function: Enzymes of unknown specificity
SACOL0872	-	3.61	3.26	-1.11	C4		↑	OsmC/Ohr family protein	Unknown function: General
SACOL1033	-	3.28	4.19	1.28	C4			hypothetical protein	-
SACOL1062	atl	3.94	4.05	1.03	C4		↑	bifunctional autolysin	Cell envelope: Biosynthesis and degradation of surface polysaccharides and lipopolysaccharides
SACOL1081	purN	2.91	1.70	-1.71	C4			phosphoribosylglycinamide formyltransferase	Purines, pyrimidines, nucleosides, and nucleotides: Purine ribonucleotide biosynthesis
SACOL1082	purH	2.52	1.68	-1.50	C4			phosphoribosylaminoimidazolecarboxamide formyltransferase/IMP cyclohydrolase	Purines, pyrimidines, nucleosides, and nucleotides: Purine ribonucleotide biosynthesis
SACOL1108	-	2.53	3.76	1.49	C4			spermidine/putrescine ABC transporter, ATP-binding protein	Transport and binding proteins: Amino acids, peptides and amines
SACOL1109	-	2.82	4.07	1.44	C4			spermidine/putrescine ABC transporter, permease protein	Transport and binding proteins: Amino acids, peptides and amines
SACOL1110	-	2.62	3.53	1.35	C4			spermidine/putrescine ABC transporter, permease protein	Transport and binding proteins: Amino acids, peptides and amines
SACOL1111	-	2.58	3.35	1.30	C4		↑	spermidine/putrescine ABC transporter, spermidine/putrescine-binding protein	Transport and binding proteins: Amino acids, peptides and amines
SACOL1183	-	2.84	3.24	1.14	C4			membrane protein, putative	Cell envelope: Other
SACOL1254	rpsP	3.84	3.34	-1.15	C4			ribosomal protein S16	Protein synthesis: Ribosomal proteins: synthesis and modification
SACOL1255	rimM	3.51	3.99	1.14	C4			16S rRNA processing protein RimM	Transcription: RNA processing
SACOL1256	trmD	3.42	3.96	1.16	C4			tRNA (guanine-N1)-methyltransferase	Protein synthesis: tRNA and rRNA base modification
SACOL1292	rpsO	2.27	2.61	1.15	C4			ribosomal protein S15	Protein synthesis: Ribosomal proteins: synthesis and modification
SACOL1371	guaC	4.01	1.95	-2.06	C4			GMP reductase	Purines, pyrimidines, nucleosides, and nucleotides: Nucleotide and nucleoside interconversions
SACOL1418	-	2.78	1.66	-1.68	C4			conserved hypothetical protein	Hypothetical proteins: Conserved
SACOL1420	-	2.52	2.72	1.08	C4			phosphate transport system protein PhoU, putative	Transport and binding proteins: Anions
SACOL1421	-	2.86	2.75	-1.04	C4			phosphate ABC transporter, ATP-binding protein	Transport and binding proteins: Anions
SACOL1422	-	3.14	3.67	1.17	C4			phosphate ABC transporter, permease protein	Transport and binding proteins: Anions
SACOL1451	arlR	1.95	2.62	1.34	C4			DNA-binding response regulator ArlR	Cellular processes: Pathogenesis; Regulatory functions: DNA interactions; Regulatory functions: Protein interactions; Signal transduction: Two-component systems
SACOL1453	murG	2.88	1.80	-1.60	C4		↑	UDP-N-acetylglucosamine--N-acetylmuramyl-(pentapeptide) pyrophosphoryl-undeca	Cell envelope: Biosynthesis and degradation of murein sacculus and peptidoglycan
SACOL1550	-	3.69	2.66	-1.38	C4			transcriptional regulator, AraC family	Regulatory functions: DNA interactions
SACOL1576	-	2.05	2.64	1.28	C4		↑	traG protein, putative	Mobile and extrachromosomal element functions: Plasmid functions
SACOL1577	-	2.15	2.71	1.27	C4			conserved hypothetical protein	Hypothetical proteins: Conserved
SACOL1700	rpmA	2.07	2.92	1.41	C4			ribosomal protein L27	Protein synthesis: Ribosomal proteins: synthesis and modification
SACOL1701	-	2.14	3.02	1.41	C4			conserved hypothetical protein	Hypothetical proteins: Conserved
SACOL1769	rpsD	3.54	2.57	-1.37	C4			ribosomal protein S4	Protein synthesis: Ribosomal proteins: synthesis and modification
SACOL1778	tyrS	2.70	1.59	-1.70	C4			tyrosyl-tRNA synthetase	Protein synthesis: tRNA aminoacylation
SACOL1871	epiG	3.69	1.91	-1.93	C4	σ^B		epidermin immunity protein F	Cellular processes: Toxin production and resistance
SACOL1872	epiE	3.32	2.24	-1.48	C4	σ^B		epidermin immunity protein F	Cellular processes: Toxin production and resistance
SACOL1882	-	2.02	2.59	1.28	C4			hypothetical protein	-
SACOL1903	-	2.02	2.61	1.29	C4			conserved hypothetical protein	Hypothetical proteins: Conserved
SACOL1935	-	2.56	2.32	-1.10	C4			conserved hypothetical protein	Hypothetical proteins: Conserved
SACOL1939	-	2.50	2.44	-1.02	C4			phosphotyrosine protein phosphatase	Regulatory functions: Protein interactions
SACOL1952	-	3.71	6.34	1.71	C4		↓	ferritins family protein	Transport and binding proteins: Cations and iron carrying compounds
SACOL2012	-	3.76	2.74	-1.38	C4			acetyltransferase, GNAT family	Unknown function: Enzymes of unknown specificity
SACOL2013	-	3.66	2.73	-1.34	C4			hypothetical protein	-
SACOL2019	-	3.81	3.27	-1.17	C4		↓	sdrH protein, putative	Cell envelope: Other
SACOL2067	kdpB	2.78	3.86	1.39	C4			K+-transporting ATPase, B subunit	Transport and binding proteins: Cations and iron carrying compounds
SACOL2131	-	3.48	6.44	1.85	C4			Dps family protein	Cellular processes: Adaptations to atypical conditions
SACOL2137	czrA	6.47	7.32	1.13	C4			transcriptional regulator CzrA	Regulatory functions: DNA interactions
SACOL2138	-	6.73	7.85	1.17	C4			cation efflux family protein	Transport and binding proteins: Cations and iron carrying compounds
SACOL2176	opuD2	3.84	2.21	-1.74	C4	σ^B	↑	osmoprotectant transporter, BCCT family	Transport and binding proteins: Amino acids, peptides and amines
SACOL2197	-	9.21	11.87	1.29	C4	σ^B	↑	surface protein, putative	Cell envelope: Other
SACOL2287	sarR	2.19	2.54	1.16	C4			staphylococcal accessory regulator R	Cellular processes: Toxin production and resistance; Regulatory functions: Other; Cellular processes: Pathogenesis; Regulatory functions: DNA interactions
SACOL2295	-	5.46	7.21	1.32	C4			staphyloxanthin biosynthesis protein, putative	Cellular processes: Pathogenesis
SACOL2398	nirB	2.76	2.37	-1.16	C4			nitrite reductase [NAD(P)H], large subunit	Central intermediary metabolism: Nitrogen metabolism
SACOL2434	-	3.04	3.17	1.04	C4	σ^B	↑	membrane protein, putative	Cell envelope: Other
SACOL2477	-	2.58	1.79	-1.44	C4			conserved hypothetical protein	Hypothetical proteins: Conserved
SACOL2481	-	3.38	2.24	-1.51	C4			hypothetical protein	-
SACOL2510	-	1.91	2.77	1.45	C4			hypothetical protein	-
SACOL2511	fnbA	2.97	5.44	1.83	C4			fibronectin-binding protein A	Cell envelope: Other; Cellular processes: Pathogenesis
SACOL2554	-	2.82	1.74	-1.62	C4	σ^B		membrane protein, putative	Cell envelope: Other
SACOL2557	-	4.33	2.64	-1.64	C4			conserved domain protein	Hypothetical proteins: Domain

SACOL2631	-	2.50	2.53	1.01	C4		conserved hypothetical protein	Hypothetical proteins: Conserved
SACOL2735	-	4.15	4.75	1.14	C4		chromosome partioning protein, ParB family	Cellular processes: Cell division
SACOL2736	gidB	3.18	4.03	1.27	C4		glucose-inhibited division protein B	Unknown function: General
SACOL2737	gidA	3.57	4.33	1.21	C4		glucose inhibited division protein A	Unknown function: General
SACOL2738	trmE	3.56	4.00	1.13	C4		tRNA modification GTPase TrmE	Cellular processes: Detoxification
SACOL2739	rnpA	3.52	4.50	1.28	C4		ribonuclease P protein component	Transcription: RNA processing
SACOL2740	rpmH	2.44	2.85	1.17	C4		ribosomal protein L34	Protein synthesis: Ribosomal proteins: synthesis and modification
SACOL0024	sasH	-3.45	-4.89	-1.42	C5		5-nucleotidase family protein	Purines, pyrimidines, nucleosides, and nucleotides: Other
SACOL0032	maoC	-1.95	-2.49	-1.28	C5		MaoC domain protein	Unknown function: General
SACOL0052	-	-1.86	-2.63	-1.41	C5		glycosyl transferase, group 1 family protein	Cell envelope: Biosynthesis and degradation of surface polysaccharides and lipopolysaccharides
SACOL0058	-	-2.78	-3.05	-1.10	C5		conserved hypothetical protein	Hypothetical proteins: Conserved
SACOL0099	sirA	-3.24	-2.47	1.31	C5		iron compound ABC transporter, iron compound-binding protein SirA	Cell envelope: Other; Transport and binding proteins: Cations and iron carrying compounds
SACOL0109	-	-1.83	-2.60	-1.42	C5		hypothetical protein	-
SACOL0110	-	-2.20	-3.58	-1.63	C5		hypothetical protein	-
SACOL0192	-	-4.90	-3.19	1.54	C5		maltose ABC transporter, ATP-binding protein, putative	Transport and binding proteins: Carbohydrates, organic alcohols, and acids
SACOL0218	-	-2.46	-4.44	-1.80	C5		conserved hypothetical protein	Hypothetical proteins: Conserved
SACOL0236	-	-2.17	-3.44	-1.58	C5		4-diphosphocytidyl-2C-methyl-D-erythritol synthase, putative	Biosynthesis of cofactors, prosthetic groups, and carriers: Other
SACOL0246	lytR	-2.19	-2.86	-1.30	C5	↓	response regulator LytR	Cell envelope: Biosynthesis and degradation of murein sacculus and peptidoglycan: Pathogenesis; Regulatory functions; Signal transduction: Two-component systems
SACOL0284	-	-3.08	-4.38	-1.42	C5		conserved hypothetical protein	Hypothetical proteins: Conserved
SACOL0297	-	-1.94	-2.67	-1.38	C5		conserved hypothetical protein	Hypothetical proteins: Conserved
SACOL0302	brnQ2	-3.68	-2.70	1.36	C5		branched-chain amino acid transport system II carrier protein	Transport and binding proteins: Amino acids, peptides and amines
SACOL0303	-	-5.88	-14.78	-2.51	C5		acid phosphatase5-nucleotidase, lipoprotein e(P4) family	Transport and binding proteins: Other; Biosynthesis of cofactors, prosthetic groups, and carriers: Pyridine nucleotides
SACOL0417	-	-4.54	-2.67	1.70	C5		MttB family protein	Protein fate: Protein and peptide secretion and trafficking
SACOL0418	-	-4.15	-2.46	1.69	C5		mttA/Hcf106 family protein	Protein fate: Protein and peptide secretion and trafficking
SACOL0419	-	-5.67	-2.79	2.03	C5		hypothetical protein	-
SACOL0512	-	-3.72	-2.12	1.75	C5		conserved hypothetical protein	Hypothetical proteins: Conserved
SACOL0553	tilS	-2.53	-1.74	1.46	C5		tRNA(Ile)-lysidine synthetase	Protein synthesis: tRNA and rRNA base modification
SACOL0566	nupC	-2.75	-3.30	-1.20	C5	↓	nucleoside permease NupC	Transport and binding proteins: Nucleosides, purines and pyrimidines
SACOL0604	-	-2.74	-2.50	1.09	C5	↓	deoxynucleoside kinase family protein	Purines, pyrimidines, nucleosides, and nucleotides: Nucleotide and nucleoside interconversions
SACOL0649	-	-2.65	-1.87	1.41	C5		conserved hypothetical protein	Hypothetical proteins: Conserved
SACOL0650	-	-2.51	-1.84	1.36	C5		conserved hypothetical protein	Hypothetical proteins: Conserved
SACOL0651	-	-4.20	-2.18	1.93	C5		conserved hypothetical protein	Hypothetical proteins: Conserved
SACOL0653	-	-2.91	-1.70	1.71	C5		conserved hypothetical protein	Hypothetical proteins: Conserved
SACOL0688	-	-4.65	-9.59	-2.06	C5		ABC transporter, substrate-binding protein	Transport and binding proteins: Unknown substrate
SACOL0689	-	-7.06	-9.80	-1.39	C5	↓	ABC transporter, permease protein	Transport and binding proteins: Unknown substrate
SACOL0690	-	-5.84	-10.93	-1.87	C5	↓	ABC transporter, ATP-binding protein	Transport and binding proteins: Unknown substrate
SACOL0693	tagA	-2.91	-2.37	1.23	C5	↓	UDP-N-acetyl-D-mannosamine transferase	Cell envelope: Biosynthesis and degradation of murein sacculus and peptidoglycan
SACOL0815	-	-3.34	-6.41	-1.92	C5		ribosomal subunit interface protein	Protein synthesis: Translation factors
SACOL0860	nuc	-5.73	-14.78	-2.58	C5	↓	thermonuclease precursor	DNA metabolism: Degradation of DNA
SACOL0887	sei	-1.98	-3.12	-1.58	C5		staphylococcal enterotoxin type I	Cellular processes: Toxin production and resistance; Cellular processes: Pathogenesis
SACOL0889	-	-2.24	-2.59	-1.16	C5		pathogenicity island protein	Cellular processes: Pathogenesis
SACOL0922	-	-2.85	-3.10	-1.09	C5	↑	conserved hypothetical protein	Hypothetical proteins: Conserved
SACOL0924	-	-2.73	-2.96	-1.08	C5		conserved hypothetical protein	Hypothetical proteins: Conserved
SACOL0930	-	-2.62	-2.79	-1.07	C5	↓	conserved hypothetical protein	Hypothetical proteins: Conserved
SACOL0959	-	-4.22	-3.21	1.31	C5		NADH-dependent flavin oxidoreductase, Oye family	Unknown function: Enzymes of unknown specificity
SACOL0975	cdr	-2.15	-3.38	-1.57	C5		CoA-disulfide reductase	Energy metabolism: Electron transport
SACOL0976	-	-2.44	-3.45	-1.41	C5		hydrolase, haloacid dehalogenase-like family	Unknown function: Enzymes of unknown specificity
SACOL1005	pepF	-2.88	-3.03	-1.05	C5		oligoendopeptidase F	Protein fate: Degradation of proteins, peptides, and glycopeptides
SACOL1028	htrA	-1.76	-2.58	-1.46	C5		serine protease	Protein fate: Degradation of proteins, peptides, and glycopeptides; Cellular processes: Pathogenesis
SACOL1034	-	-2.90	-4.32	-1.49	C5		lipoate-protein ligase A family protein	Protein fate: Protein modification and repair
SACOL1048	-	-3.30	-2.58	1.28	C5	↓	acetyltransferase, GNAT family	Unknown function: Enzymes of unknown specificity
SACOL1094	cydA	-2.81	-2.19	1.28	C5		cytochrome d ubiquinol oxidase, subunit I	Energy metabolism: Electron transport
SACOL1095	cydB	-2.75	-2.15	1.28	C5		cytochrome d ubiquinol oxidase, subunit II	Energy metabolism: Electron transport
SACOL1115	-	-2.32	-3.53	-1.52	C5		conserved hypothetical protein	Hypothetical proteins: Conserved
SACOL1140	sasE	-5.88	-5.72	1.03	C5		LPXTG cell wall surface anchor protein	Cell envelope: Other
SACOL1142	isdD	-2.74	-1.71	1.60	C5		hypothetical protein	-
SACOL1143	-	-2.78	-1.73	1.60	C5		iron compound ABC transporter, iron compound-binding protein, putative	Cell envelope: Other; Transport and binding proteins: Cations and iron carrying compounds
SACOL1145	srtB	-2.56	-2.78	-1.09	C5		sortase B	Cell envelope: Other
SACOL1150	rnhC	-2.20	-2.49	-1.13	C5		ribonuclease HIII	DNA metabolism: DNA replication, recombination, and repair
SACOL1206	ileS	-2.46	-3.72	-1.52	C5		isoleucyl-tRNA synthetase	Protein synthesis: tRNA aminoacylation
SACOL1296	-	-3.37	-2.77	1.22	C5		transcriptional regulator, GntR family, putative	Regulatory functions: DNA interactions
SACOL1297	-	-5.26	-3.06	1.72	C5		conserved hypothetical protein	Hypothetical proteins: Conserved
SACOL1298	-	-3.04	-4.03	-1.33	C5		peptidase, M16 family	Protein fate: Degradation of proteins, peptides, and glycopeptides
SACOL1299	-	-2.85	-4.16	-1.46	C5		acetoacetyl-CoA reductase, putative	Fatty acid and phospholipid metabolism: Biosynthesis
SACOL1300	-	-2.34	-3.87	-1.65	C5		ACT domain protein	Unknown function: General
SACOL1321	glpD	-2.05	-2.90	-1.42	C5		aerobic glycerol-3-phosphate dehydrogenase	Energy metabolism: Other
SACOL1328	glnR	-2.34	-3.00	-1.28	C5		glutamine synthetase repressor	Cellular processes: Toxin production and resistance; Regulatory functions: DNA interactions
SACOL1419	-	-10.64	-11.53	-1.08	C5		oligoendopeptidase F, putative	Protein fate: Degradation of proteins, peptides, and glycopeptides
SACOL1438	-	-5.99	-5.02	1.19	C5		conserved hypothetical protein	Hypothetical proteins: Conserved
SACOL1471	-	-2.77	-1.77	1.56	C5		cell wall enzyme EbsB, putative	Cell envelope: Other; Protein fate: Degradation of proteins, peptides, and glycopeptides
SACOL1481	-	-3.88	-4.73	-1.22	C5		conserved hypothetical protein	Hypothetical proteins: Conserved
SACOL1495	-	-2.06	-3.14	-1.52	C5		DNA polymerase III, epsilon subunit/ATP-dependent helicase DinG	DNA metabolism: DNA replication, recombination, and repair
SACOL1496	birA	-2.28	-2.57	-1.13	C5		birA bifunctional protein	Biosynthesis of cofactors, prosthetic groups, and carriers: Biotin; Protein fate: Protein modification and repair; Regulatory functions: DNA interactions
SACOL1530	-	-2.16	-2.54	-1.17	C5		lipoprotein, putative	Cell envelope: Other
SACOL1552	malR	-3.46	-2.28	1.51	C5	↑	maltose operon repressor	Regulatory functions: DNA interactions
SACOL1564	recN	-2.71	-3.76	-1.39	C5		DNA repair protein RecN	DNA metabolism: DNA replication, recombination, and repair
SACOL1565	argR	-3.99	-3.93	1.02	C5	↓	arginine repressor	Amino acid biosynthesis: Glutamate family; Regulatory functions: DNA interactions
SACOL1674	-	-2.47	-1.94	1.27	C5		helicase, putative, RecD/TraA family	Unknown function: Enzymes of unknown specificity
SACOL1743	-	-2.17	-2.89	-1.33	C5		amino acid permease	Transport and binding proteins: Amino acids, peptides and amines
SACOL1771	-	-2.38	-3.39	-1.42	C5		OsmC/Ohr family protein	Unknown function: General
SACOL1777	-	-3.69	-4.74	-1.29	C5		serine protease HtrA, putative	Protein fate: Degradation of proteins, peptides, and glycopeptides
SACOL1816	putA	-4.11	-2.21	1.86	C5		proline dehydrogenase	Energy metabolism: Amino acids and amines
SACOL1818	ribBA	-3.15	-2.12	1.48	C5		3,4-dihydroxy-2-butanone-4-phosphate synthase/GTP cyclohydrolase II	Biosynthesis of cofactors, prosthetic groups, and carriers: Riboflavin, FMN, and FAD
SACOL1819	ribE	-3.59	-1.96	1.83	C5		riboflavin synthase, alpha subunit	Biosynthesis of cofactors, prosthetic groups, and carriers: Riboflavin, FMN, and FAD
SACOL1820	ribD	-3.41	-1.94	1.76	C5		riboflavin biosynthesis protein RibD	Biosynthesis of cofactors, prosthetic groups, and carriers: Riboflavin, FMN, and FAD
SACOL1833	-	-2.96	-2.09	1.42	C5		crcB family protein	Unknown function: General

SACOL1838	pckA	-3.24	-2.43	1.33	C5		phosphoenolpyruvate carboxykinase (ATP)	Energy metabolism: Glycolysis/gluconeogenesis
SACOL1840	-	-3.26	-5.42	-1.66	C5		conserved hypothetical protein	Hypothetical proteins: Conserved
SACOL1841	ytkD	-4.82	-5.30	-1.10	C5		nucleoside triphosphatase YtkD	DNA metabolism: DNA replication, recombination, and repair
SACOL1844	-	-3.47	-6.46	-1.86	C5		O-succinylbenzoic acid--CoA ligase, putative	Biosynthesis of cofactors, prosthetic groups, and carriers: Menaquinone and ubiquinone
SACOL1848	-	-4.91	-5.68	-1.16	C5		lipoprotein, putative	-
SACOL1851	-	-3.42	-3.39	1.01	C5	↓	hypothetical protein	-
SACOL1893	-	-2.20	-3.67	-1.67	C5		ABC transporter, ATP-binding protein	Transport and binding proteins: Unknown substrate
SACOL1946	-	-3.35	-4.13	-1.23	C5		methionine aminopeptidase, type I	Protein fate: Protein modification and repair
SACOL1984	aldA2	-2.24	-3.14	-1.40	C5		aldehyde dehydrogenase	Energy metabolism: Fermentation
SACOL1992	-	-2.60	-2.19	1.19	C5		conserved hypothetical protein	Hypothetical proteins: Conserved
SACOL2017	groES	-2.11	-2.79	-1.32	C5		chaperonin, 10 kDa	Protein fate: Protein folding and stabilization
SACOL2075	ftsW	-2.53	-2.73	-1.08	C5		cell division protein, FtsW/RodA/SpoVE family	Cellular processes: Cell division
SACOL2130	deoD2	-2.24	-3.23	-1.44	C5		purine nucleoside phosphorylase	Purines, pyrimidines, nucleosides, and nucleotides: Salvage of nucleosides and nucleotides
SACOL2146	-	-3.42	-5.27	-1.54	C5		PTS system, mannitol-specific IIBC components	Transport and binding proteins: Carbohydrates, organic alcohols, and acids; Signal transduction: PTS
SACOL2164	-	-4.24	-3.86	1.10	C5		conserved hypothetical protein	Hypothetical proteins: Conserved
SACOL2179	-	-2.05	-2.97	-1.44	C5		conserved hypothetical protein	Hypothetical proteins: Conserved
SACOL2193	-	-3.04	-4.85	-1.59	C5		transcriptional regulator, MerR family	Regulatory functions: DNA interactions
SACOL2273	fdhD	-3.30	-1.84	1.80	C5		formate dehydrogenase accessory protein FdhD	Energy metabolism: Anaerobic; Energy metabolism: Electron transport
SACOL2280	ureA	-2.95	-3.11	-1.05	C5		urease, gamma subunit	Central intermediary metabolism: Nitrogen metabolism
SACOL2281	ureB	-3.19	-2.88	1.11	C5		urease, beta subunit	Central intermediary metabolism: Nitrogen metabolism
SACOL2306	-	-4.23	-5.94	-1.41	C5		abortive infection protein family	Unknown function: General
SACOL2310	-	-2.36	-2.73	-1.16	C5		conserved hypothetical protein	Hypothetical proteins: Conserved
SACOL2311	-	-1.75	-2.49	-1.43	C5		hypothetical protein	-
SACOL2362	mqo1	-2.70	-5.06	-1.87	C5		malate:quinone oxidoreductase	Energy metabolism: TCA cycle
SACOL2470	-	-2.64	-3.35	-1.27	C5		hypothetical protein	-
SACOL2486	-	-2.34	-4.15	-1.77	C5		hypothetical protein	-
SACOL2487	-	-3.35	-3.47	-1.03	C5		conserved hypothetical protein	Hypothetical proteins: Conserved
SACOL2488	-	-2.81	-3.33	-1.18	C5		oxidoreductase, short-chain dehydrogenase/reductase family	Unknown function: Enzymes of unknown specificity
SACOL2491	-	-2.93	-4.08	-1.39	C5		conserved hypothetical protein	Hypothetical proteins: Conserved
SACOL2501	-	-2.63	-4.77	-1.81	C5		phosphoglucomutase/phosphomannomutase family protein	Energy metabolism: Sugars
SACOL2502	-	-4.45	-3.18	1.40	C5		hypothetical protein	-
SACOL2503	-	-3.47	-2.87	1.21	C5		hypothetical protein	-
SACOL2518	-	-3.77	-5.19	-1.38	C5		conserved hypothetical protein	Hypothetical proteins: Conserved
SACOL2522	-	-3.05	-2.40	1.27	C5		DedA family protein	Unknown function: General
SACOL2551	-	-2.48	-2.53	-1.02	C5		conserved hypothetical protein TIGR00051	Hypothetical proteins: Conserved
SACOL2606	pyrD	-2.68	-1.97	1.36	C5	↓	dihydroorotate dehydrogenase	Purines, pyrimidines, nucleosides, and nucleotides: Pyrimidine ribonucleotide biosynthesis
SACOL2660	isaB	-2.72	-4.70	-1.73	C5		immunodominant antigen B	Unknown function: General
SACOL0003	-	-1.49	-2.80	-1.88	C6		conserved hypothetical protein	Hypothetical proteins: Conserved
SACOL0004	-	-1.61	-2.68	-1.66	C6	↓	recF protein	DNA metabolism: DNA replication, recombination, and repair
SACOL0005	gyrB	-1.57	-2.59	-1.65	C6		DNA gyrase, B subunit	DNA metabolism: DNA replication, recombination, and repair
SACOL0006	gyrA	-1.76	-3.93	-2.23	C6		DNA gyrase, A subunit	DNA metabolism: DNA replication, recombination, and repair
SACOL0009	serS	-2.11	-3.72	-1.77	C6		seryl-tRNA synthetase	Protein synthesis: tRNA aminoacylation
SACOL0023	yycJ	-1.47	-3.01	-2.04	C6		metallo-beta-lactamase family protein YycJ	Unknown function: General
SACOL0025	-	-1.45	-3.22	-2.23	C6		conserved hypothetical protein	Hypothetical proteins: Conserved
SACOL0173	ipdC	-1.35	-3.80	-2.80	C6		indole-3-pyruvate decarboxylase	Central intermediary metabolism: Other
SACOL0181	-	-1.62	-3.09	-1.90	C6	↓	conserved domain protein	Hypothetical proteins: Domain
SACOL0182	-	-2.13	-4.50	-2.11	C6		hypothetical protein	-
SACOL0183	-	-1.57	-2.65	-1.69	C6		hypothetical protein	-
SACOL0219	-	-2.38	-9.53	-4.00	C6		hypothetical protein	-
SACOL0220	-	-1.74	-3.62	-2.08	C6		flavohemoprotein, putative	Unknown function: General
SACOL0225	-	-2.50	-4.95	-1.98	C6		inosine-uridine preferring nucleoside hydrolase	Purines, pyrimidines, nucleosides, and nucleotides: Salvage of nucleosides and nucleotides
SACOL0274	-	-1.71	-6.28	-3.68	C6		conserved hypothetical protein	Hypothetical proteins: Conserved
SACOL0275	-	-1.60	-6.37	-3.98	C6		conserved hypothetical protein	Hypothetical proteins: Conserved
SACOL0299	-	-1.30	-2.69	-2.07	C6	↑	lipoprotein, putative	-
SACOL0477	-	-1.72	-3.55	-2.06	C6		type I restriction-modification system, S subunit, EcoA family, putative	DNA metabolism: Restriction/modification
SACOL0478	-	-2.39	-4.32	-1.80	C6		exotoxin 3, putative	Cellular processes: Toxin production and resistance
SACOL0536	ksgA	-1.50	-2.54	-1.69	C6		dimethyladenosine transferase	Cellular processes: Toxin production and resistance; Protein synthesis: tRNA and rRNA base modification
SACOL0558	folP	-1.96	-4.08	-2.08	C6		dihydropteroate synthase	Biosynthesis of cofactors, prosthetic groups, and carriers: Folic acid
SACOL0564	-	-1.37	-4.28	-3.11	C6	↓	pyridoxine biosynthesis protein	Biosynthesis of cofactors, prosthetic groups, and carriers: Pyridoxine
SACOL0567	ctsR	-1.68	-2.63	-1.57	C6		transcriptional regulator CtsR	Regulatory functions: DNA interactions
SACOL0602	-	-1.35	-2.48	-1.83	C6		hydrolase, haloacid dehalogenase-like family	Unknown function: Enzymes of unknown specificity
SACOL0633	-	-1.71	-5.11	-3.00	C6		conserved hypothetical protein	Hypothetical proteins: Conserved
SACOL0714	-	-1.64	-2.84	-1.73	C6		acetyltransferase, GNAT family	Unknown function: Enzymes of unknown specificity
SACOL0739	-	-1.49	-3.61	-2.42	C6	↑	acetyltransferase, GNAT family	Unknown function: Enzymes of unknown specificity
SACOL0781	-	-1.37	-2.82	-2.07	C6	↑	osmoprotectant ABC transporter, ATP-binding protein, putative	Transport and binding proteins: Amino acids, peptides and amines
SACOL0799	-	-1.41	-2.74	-1.94	C6		transferrin receptor	Cell envelope: Other; Transport and binding proteins: Cations and iron carrying compounds
SACOL0833	clpP	-1.35	-3.35	-2.48	C6		ATP-dependent Clp protease, proteolytic subunit ClpP	Protein fate: Degradation of proteins, peptides, and glycopeptides
SACOL0849	-	-1.30	-4.61	-3.55	C6		hypothetical protein	-
SACOL0850	-	-1.49	-3.03	-2.03	C6		hypothetical protein	-
SACOL0851	-	-2.35	-4.89	-2.08	C6		lipoprotein, putative	-
SACOL0859	-	-1.88	-3.11	-1.65	C6	↓	hypothetical protein	-
SACOL0875	-	-1.24	-3.21	-2.59	C6		thioredoxin, putative	Energy metabolism: Electron transport
SACOL0876	-	-1.56	-3.80	-2.44	C6		arsenate reductase, putative	Cellular processes: Detoxification
SACOL0877	gcvH	-1.35	-2.90	-2.16	C6		glycine cleavage system H protein	Energy metabolism: Amino acids and amines
SACOL0888	-	-1.91	-3.39	-1.78	C6		pathogenicity island, lipoprotein, putative	Cell envelope: Other; Cellular processes: Pathogenesis
SACOL0925	-	-1.66	-2.69	-1.63	C6		conserved hypothetical protein	Hypothetical proteins: Conserved
SACOL0926	-	-1.43	-2.98	-2.08	C6		5-nucleotidase family protein	Purines, pyrimidines, nucleosides, and nucleotides: Other
SACOL0931	-	-1.62	-2.74	-1.70	C6		hydrolase, haloacid dehalogenase-like family	Unknown function: Enzymes of unknown specificity
SACOL0944	-	-1.52	-3.49	-2.30	C6	↑	NADH dehydrogenase, putative	Energy metabolism: Electron transport
SACOL0977	-	-1.52	-2.88	-1.89	C6		conserved hypothetical protein	Hypothetical proteins: Conserved
SACOL0987	fabH	-2.19	-4.48	-2.04	C6		3-oxoacyl-(acyl-carrier-protein) synthase III	Fatty acid and phospholipid metabolism: Biosynthesis
SACOL0988	fabF	-1.53	-3.31	-2.16	C6		3-oxoacyl-(acyl-carrier-protein) synthase II	Fatty acid and phospholipid metabolism: Biosynthesis
SACOL1168	efb	-1.36	-4.43	-3.25	C6		fibrinogen-binding protein	Cell envelope: Other
SACOL1301	-	-1.77	-3.23	-1.82	C6	↑	transcriptional regulator, putative	Regulatory functions: Other
SACOL1302	pgsA	-1.72	-3.66	-2.13	C6		CDP-diacylglycerol--glycerol-3-phosphate 3-phosphatidyltransferase	Fatty acid and phospholipid metabolism: Biosynthesis
SACOL1325	gpxA1	-1.46	-2.58	-1.77	C6		glutathione peroxidase	Biosynthesis of cofactors, prosthetic groups, and carriers: Glutathione and analogs; Cellular processes: Detoxification

SACOL1365	-	-1.23	-2.58	-2.09	C6			hydrolase, haloacid dehalogenase-like family	Unknown function: Enzymes of unknown specificity
SACOL1377	tkt	-1.69	-2.54	-1.50	C6			transketolase	Energy metabolism: Pentose phosphate pathway
SACOL1439	-	-1.58	-3.16	-2.01	C6			acylphosphatase	Fatty acid and phospholipid metabolism: Other
SACOL1440	-	-1.23	-3.26	-2.65	C6			xpaC protein, putative	Cellular processes: Other
SACOL1516	rpsA	-1.40	-2.92	-2.08	C6			ribosomal protein S1	Protein synthesis: Ribosomal proteins: synthesis and modification
SACOL1520	-	-1.82	-2.94	-1.62	C6			pyridine nucleotide-disulfide oxidoreductase	Unknown function: Enzymes of unknown specificity
SACOL1554	gnd	-1.44	-2.89	-2.01	C6			6-phosphogluconate dehydrogenase, decarboxylating	Energy metabolism: Pentose phosphate pathway
SACOL1562	-	-1.43	-2.62	-1.83	C6			2-oxoisovalerate dehydrogenase, E1 component, alpha subunit	Energy metabolism: Amino acids and amines
SACOL1563	lpdA	-1.52	-2.53	-1.66	C6			2-oxoisovalerate dehydrogenase, E3 component, lipoamide dehydrogenase	Energy metabolism: Amino acids and amines
SACOL1566	ispA	-1.45	-2.80	-1.94	C6			geranyltranstransferase	Biosynthesis of cofactors, prosthetic groups, and carriers: Menaquinone and ubiquinone
SACOL1568	xseA	-1.45	-2.85	-1.97	C6			exodeoxyribonuclease VII, large subunit	DNA metabolism: Degradation of DNA
SACOL1588	-	-1.31	-3.00	-2.28	C6			proline dipeptidase	Protein fate: Degradation of proteins, peptides, and glycopeptides
SACOL1629	-	-1.22	-2.49	-2.04	C6			conserved hypothetical protein	Hypothetical proteins: Conserved
SACOL1630	-	-1.58	-2.55	-1.62	C6			conserved hypothetical protein	Hypothetical proteins: Conserved
SACOL1631	-	-1.71	-2.82	-1.65	C6			conserved hypothetical protein	Hypothetical proteins: Conserved
SACOL1714	hemL1	-1.28	-3.26	-2.55	C6			glutamate-1-semialdehyde-2,1-aminomutase	Biosynthesis of cofactors, prosthetic groups, and carriers: Heme, porphyrin, and cobalamin
SACOL1715	hemB	-1.49	-3.19	-2.14	C6			delta-aminolevulinic acid dehydratase	Biosynthesis of cofactors, prosthetic groups, and carriers: Heme, porphyrin, and cobalamin
SACOL1716	hemD	-1.75	-3.06	-1.75	C6			uroporphyrinogen-III synthase	Biosynthesis of cofactors, prosthetic groups, and carriers: Heme, porphyrin, and cobalamin
SACOL1782	fhs	-1.62	-5.16	-3.18	C6			formate--tetrahydrofolate ligase	Central intermediary metabolism: One-carbon metabolism
SACOL1835	-	-1.53	-2.61	-1.71	C6			oxidoreductase, aldo/keto reductase family	Unknown function: Enzymes of unknown specificity
SACOL1843	-	-1.94	-4.00	-2.06	C6			o-succinylbenzoic acid (OSB) synthetase, putative	Biosynthesis of cofactors, prosthetic groups, and carriers: Menaquinone and ubiquinone
SACOL1892	-	-1.43	-3.36	-2.35	C6			membrane protein, putative	Cell envelope: Other
SACOL1943	vraS	-1.40	-2.68	-1.91	C6			sensor histidine kinase VraS	Cellular processes: Toxin production and resistance; Regulatory functions: Protein interactions; Signal transduction: Two-component systems
SACOL1961	gatA	-1.28	-2.64	-2.07	C6			glutamyl-tRNA(Gln) amidotransferase, A subunit	Protein synthesis: tRNA aminoacylation
SACOL1970	sspB2	-1.36	-2.56	-1.88	C6		↓	cysteine protease precursor SspB	Protein fate: Degradation of proteins, peptides, and glycopeptides; Cellular processes: Pathogenesis
SACOL2018	-	-2.78	-6.45	-2.32	C6		↓	abortive infection protein family	Unknown function: General
SACOL2126	luxS	-1.42	-2.82	-1.99	C6			autoinducer-2 production protein LuxS	Cellular processes: Other
SACOL2186	-	-1.28	-2.88	-2.24	C6			galactose-6-phosphate isomerase, LacA subunit	Energy metabolism: Biosynthesis and degradation of polysaccharides
SACOL2241	-	-2.84	-6.72	-2.37	C6			conserved hypothetical protein	Hypothetical proteins: Conserved
SACOL2439	-	-2.36	-4.55	-1.93	C6			lipoprotein, putative	Cell envelope: Other
SACOL2534	frp	-1.74	-2.98	-1.71	C6			NAD(P)H-flavin oxidoreductase	Energy metabolism: Electron transport
SACOL2535	-	-1.71	-2.72	-1.59	C6			D-isomer specific 2-hydroxyacid dehydrogenase family protein	Central intermediary metabolism: Other
SACOL2592	-	-1.75	-3.54	-2.01	C6		↑	conserved hypothetical protein	Hypothetical proteins: Conserved
SACOL2593	-	-1.81	-3.30	-1.82	C6		↑	transcriptional regulator, TetR family	Regulatory functions: DNA interactions
SACOL2618	ldh2	-1.95	-5.19	-2.67	C6			L-lactate dehydrogenase	Energy metabolism: Anaerobic; Energy metabolism: Glycolysis/gluconeogenesis
SACOL2688	icaR	-3.14	-8.59	-2.74	C6			intercellular adhesion regulator	Cellular processes: Pathogenesis; Regulatory functions: DNA interactions
SACOL2731	-	-2.58	-5.59	-2.17	C6			cold shock protein, CSD family	Cellular processes: Adaptations to atypical conditions
SACOL0170	rocD1	-1.48	2.42	3.57	C7			ornithine aminotransferase	Energy metabolism: Amino acids and amines
SACOL0430	-	-1.57	1.61	2.53	C7		↓	trans-sulfuration enzyme family protein	Amino acid biosynthesis: Aspartate family
SACOL0494	nuoF	-2.82	-1.33	2.12	C7			NADH dehydrogenase I, F subunit	Energy metabolism: Electron transport
SACOL0501	-	-8.34	-2.18	3.83	C7			sodium-dependent transporter, putative	Transport and binding proteins: Unknown substrate; Transport and binding proteins: Cations and iron carrying compounds
SACOL0502	-	-2.26	2.98	6.74	C7			cysteine synthase/cystathionine beta-synthase family protein	Amino acid biosynthesis: Serine family
SACOL0506	-	-1.25	2.28	2.85	C7			ABC transporter, substrate-binding protein	Transport and binding proteins: Unknown substrate
SACOL0516	-	-2.04	1.38	2.83	C7			PTS system, IIBC components	Transport and binding proteins: Carbohydrates, organic alcohols, and acids; Signal transduction: PTS
SACOL0621	-	-1.76	1.74	3.06	C7			substrate--CoA ligase, putative	Fatty acid and phospholipid metabolism: Other
SACOL0652	-	-2.55	-1.25	2.05	C7			conserved hypothetical protein	Hypothetical proteins: Conserved
SACOL0725	-	-2.57	1.26	3.23	C7			transcriptional regulator, AraC family	Regulatory functions: DNA interactions
SACOL0769	-	-1.84	1.61	2.95	C7			conserved hypothetical protein	Hypothetical proteins: Conserved
SACOL1044	-	-2.54	-1.44	1.77	C7			conserved hypothetical protein	Hypothetical proteins: Conserved
SACOL1141	isdC	-2.99	-1.45	2.06	C7			NPQTN cell wall surface anchor protein	Cell envelope: Other
SACOL1269	xerC	-2.24	1.18	2.64	C7			tyrosine recombinase XerC	DNA metabolism: DNA replication, recombination, and repair
SACOL1675	-	-2.73	-1.58	1.73	C7		↓	TPR domain protein	Unknown function: General
SACOL1687	-	-2.68	-1.52	1.76	C7			N-acetylmuramoyl-L-alanine amidase, family 3	Cell envelope: Biosynthesis and degradation of surface polysaccharides and lipopolysaccharides
SACOL1832	crcB	-2.50	-1.32	1.89	C7			crcB protein	Unknown function: General
SACOL1904	-	-1.48	1.79	2.65	C7		↓	transcriptional regulator, putative	Regulatory functions: Other
SACOL2086	-	-2.77	1.37	3.81	C7			transcriptional regulator, TenA family	Regulatory functions: Other
SACOL2157	-	-1.35	1.87	2.52	C7			drug resistance transporter, EmrB/QacA subfamily	Cellular processes: Toxin production and resistance; Transport and binding proteins: Other
SACOL2158	-	-2.37	1.15	2.74	C7			conserved hypothetical protein	Hypothetical proteins: Conserved
SACOL2159	-	-1.48	1.92	2.83	C7			drug transporter, putative	Cellular processes: Toxin production and resistance; Transport and binding proteins: Other
SACOL2163	-	-2.90	-1.39	2.09	C7			conserved hypothetical protein	Hypothetical proteins: Conserved
SACOL2440	-	-2.73	-1.56	1.75	C7			conserved hypothetical protein	Hypothetical proteins: Conserved
SACOL2463	pepA2	-1.39	3.00	4.17	C7			glutamyl-aminopeptidase	Protein fate: Degradation of proteins, peptides, and glycopeptides
SACOL2509	fnbB	-2.01	2.90	5.83	C7			fibronectin binding protein B	Cell envelope: Other
SACOL2689	icaA	-1.42	2.53	3.60	C7			intercellular adhesion protein A	Cell envelope: Biosynthesis and degradation of surface polysaccharides and lipopolysaccharides
SACOL2690	icaD	-1.47	2.14	3.13	C7			intercellular adhesion protein D	Cell envelope: Biosynthesis and degradation of surface polysaccharides and lipopolysaccharides
SACOL0018	purA	1.87	-2.17	-4.06	C8			adenylosuccinate synthetase	Purines, pyrimidines, nucleosides, and nucleotides: Purine ribonucleotide biosynthesis
SACOL0026	-	-1.15	-3.07	-2.67	C8			conserved hypothetical protein	Hypothetical proteins: Conserved
SACOL0033	mecA	1.26	-1.98	-2.49	C8			penicillin-binding protein 2	Cell envelope: Biosynthesis and degradation of murein sacculus and peptidoglycan
SACOL0035	-	1.77	-1.88	-3.33	C8			hypothetical protein	-
SACOL0092	-	1.61	-2.20	-3.55	C8		↑	conserved hypothetical protein	Hypothetical proteins: Conserved
SACOL0095	spa	1.10	-5.78	-6.35	C8			immunoglobulin G binding protein A precursor	Cellular processes: Pathogenesis
SACOL0111	-	-1.14	-2.57	-2.25	C8			acetoin reductase	Energy metabolism: Fermentation
SACOL0119	sasD	1.25	-2.24	-2.81	C8			cell wall surface anchor family protein	Cell envelope: Other
SACOL0276	yukA	1.02	-2.43	-2.48	C8			diarrheal toxin	Unknown function: General
SACOL0300	-	-1.06	-2.93	-2.75	C8			hypothetical protein	-
SACOL0565	-	-1.18	-2.86	-2.43	C8		↓	conserved hypothetical protein	Hypothetical proteins: Conserved
SACOL0617	-	1.02	-2.67	-2.72	C8	σ^B	↑	hexulose-6-phosphate synthase, putative	Energy metabolism: Sugars
SACOL0618	-	1.20	-2.20	-2.64	C8	σ^B	↑	SIS domain protein	Unknown function: General
SACOL0635	-	1.64	-1.64	-2.69	C8			lipoate-protein ligase A family protein	Protein fate: Protein modification and repair
SACOL0763	-	2.08	-2.74	-5.70	C8		↑	oxidoreductase, aldo/keto reductase family	Unknown function: Enzymes of unknown specificity
SACOL0777	-	1.11	-2.39	-2.66	C8			urea amidolyase-related protein	Unknown function: General
SACOL0778	-	1.64	-1.51	-2.48	C8			sulfatase family protein	Unknown function: Enzymes of unknown specificity
SACOL0783	opuBB	1.26	-2.06	-2.59	C8		↑	osmoprotectant ABC transporter, permease protein	Transport and binding proteins: Amino acids, peptides and amines
SACOL0957	-	1.61	-2.32	-3.74	C8		↓	peptidyl-prolyl cis-trans isomerase, cyclophilin-type	Protein fate: Protein folding and stabilization
SACOL0985	-	1.42	-2.57	-3.65	C8			surface protein, putative	Cell envelope: Other
SACOL1123	pyc	1.13	-2.24	-2.53	C8		↓	pyruvate carboxylase	Energy metabolism: Glycolysis/gluconeogenesis

SACOL1164	-	1.91	**-3.35**	**-6.40**	**C8**			fibrinogen binding-related protein	Unknown function: General
SACOL1169	-	-1.14	**-4.32**	**-3.80**	**C8**			fibrinogen-binding protein precursor-related protein	Unknown function: General
SACOL1317	glpP	-1.07	**-2.61**	**-2.44**	**C8**			glycerol uptake operon antiterminator regulatory protein	Regulatory functions: RNA interactions
SACOL1329	femC	-1.05	**-3.56**	**-3.38**	**C8**			glutamine synthetase FemC	Cell envelope: Biosynthesis and degradation of murein sacculus and peptidoglycan
SACOL1351	cls1	-1.05	**-2.58**	**-2.45**	**C8**			cardiolipin synthetase	Fatty acid and phospholipid metabolism: Biosynthesis
SACOL1441	-	1.16	**-3.30**	**-3.81**	**C8**			tellurite resistance protein, putative	Cellular processes: Toxin production and resistance
SACOL1456	-	**1.86**	-2.14	**-3.97**	**C8**			conserved hypothetical protein	Hypothetical proteins: Conserved
SACOL1457	-	**1.66**	**-2.83**	**-4.70**	**C8**			PTS system, IIA component	Transport and binding proteins: Carbohydrates, organic alcohols, and acids; Signal transduction: PTS
SACOL1623	-	1.20	**-2.45**	**-2.95**	**C8**			DNA repair protein RecO family	DNA metabolism: DNA replication, recombination, and repair
SACOL1670	-	**1.67**	-1.55	**-2.60**	**C8**			conserved hypothetical protein	Hypothetical proteins: Conserved
SACOL1753	-	1.22	**-2.81**	**-3.42**	**C8**			universal stress protein family	Cellular processes: Adaptations to atypical conditions
SACOL1759	-	-1.11	**-3.64**	**-3.28**	**C8**			universal stress protein family	Cellular processes: Adaptations to atypical conditions
SACOL1760	ackA	1.14	**-3.37**	**-3.83**	**C8**		↓	acetate kinase	Central intermediary metabolism: Other; Energy metabolism: Fermentation
SACOL1796	-	**1.70**	-1.89	**-3.21**	**C8**			conserved hypothetical protein	Hypothetical proteins: Conserved
SACOL1831	tal	-1.18	**-5.26**	**-4.46**	**C8**			transaldolase	Energy metabolism: Pentose phosphate pathway
SACOL1942	vraR	-1.16	**-2.69**	-2.31	**C8**			DNA-binding response regulator VraR	Cellular processes: Toxin production and resistance; Regulatory functions: DNA interactions; Regulatory functions; Signal transduction: Two-component systems
SACOL1960	gatB	1.03	**-2.44**	**-2.50**	**C8**			glutamyl-tRNA(Gln) amidotransferase, B subunit	Protein synthesis: tRNA aminoacylation
SACOL1964	camS	-1.03	**-2.60**	**-2.53**	**C8**			Staphylococcus aureus sex pheromone	Cellular processes: Other; Cellular processes: Cell adhesion
SACOL1965	ligA	-1.01	**-2.57**	**-2.54**	**C8**			DNA ligase, NAD-dependent	DNA metabolism: DNA replication, recombination, and repair
SACOL1966	pcrA	1.04	**-2.38**	**-2.49**	**C8**			ATP-dependent DNA helicase PcrA	DNA metabolism: DNA replication, recombination, and repair
SACOL1968	-	1.05	**-3.20**	**-3.34**	**C8**			conserved hypothetical protein	Hypothetical proteins: Conserved
SACOL1969	purB	1.24	**-2.94**	**-3.65**	**C8**			adenylosuccinate lyase	Purines, pyrimidines, nucleosides, and nucleotides: Purine ribonucleotide biosynthesis
SACOL2006	-	**1.61**	**-1.64**	**-2.64**	**C8**			Aerolysin/Leukocidin family protein	Cellular processes: Toxin production and resistance; Cellular processes: Pathogenesis
SACOL2117	fbaA	-1.21	**-4.13**	**-3.41**	**C8**		↓	fructose-bisphosphate aldolase, class II	Energy metabolism: Glycolysis/gluconeogenesis
SACOL2192	-	1.07	**-3.06**	**-3.28**	**C8**			oxidoreductase, aldo/keto reductase family	Unknown function: Enzymes of unknown specificity
SACOL2415	gpm	1.19	**-2.53**	**-3.01**	**C8**		↑	phosphoglycerate mutase	Energy metabolism: Glycolysis/gluconeogenesis
SACOL2418	-	-1.13	**-4.54**	**-4.03**	**C8**			IgG-binding protein SBI	Cellular processes: Pathogenesis
SACOL2467	-	**1.74**	**-1.74**	**-3.02**	**C8**		↓	lipoprotein, putative	Cell envelope: Other
SACOL2576	crtN	1.67	**-1.58**	**-2.65**	**C8**	σ^B	↑	dehydrosqualene desaturase	Central intermediary metabolism: Other
SACOL2622	fdaB	-1.09	**-8.52**	**-7.83**	**C8**		↑	fructose-bisphosphate aldolase, class I	Energy metabolism: Glycolysis/gluconeogenesis
SACOL2667	-	1.17	**-2.39**	**-2.81**	**C8**		↑	isochorismatase family protein	Unknown function: Enzymes of unknown specificity

1) Fold change: genes that passed the significance test (see materials and methods section) are shown in bold. Red: at least 2.5 fold up-regulation. Green: at least -2.5 fold down regulation.
2) Genes grouped based on expression kinetics. **C1**: up-regulated at 10 min and down regulated at 40 min; **C2**: unchanged at 10 min and up-regulated at 40 min; **C3**: up-regulated at 10 min and further up-regulated at 40 min; **C4**:
up-regulated at 10 min and remains up-regulated at a similar level at 40 min; C5: down-regulated at 10 min and remains down-regulated at a similar level at 40 min; .
regulated at 10 min and up-regulated at 40 min; C8: unchanged at 10 min and down-regulated at 40 min
3)Genes positively regulated by SigB as identified by Bischoff et al. 2004 and Pané-Farré et al. 2006 (overlap between both studies). Genes organized in transcription units that are transcribed from an experimentally confirmed SigB-dependent promoter are shaded gray (Bischoff et al. 2004, Pané-Farré et al. 2006, Homerova et al. 2004).
4) Upward and downward arrows show genes induced or repressed in response to linoleic acid based on Kenny et al. 2009.

Bischoff, M., P. Dunman, J. Kormanec, D. Macapagal, E. Murphy, W. Mounts, B. Berger-Bächi, and S. Projan. 2004. Microarray-based analysis of the Staphylococcus aureus sigmaB regulon. J. Bacteriol. 186:4085-4099.
Homerova, D., M. Bischoff, A. Dumolin,and J. Kormanec. 2004. Optimization of a two-plasmid system for the identification of promoters recognized by RNA polymerase containing Staphylococcus aureus alternative sigma factor sigmaB. FEMS Microbiol. Lett. **232**:173-9.
Kenny, J. G., D. Ward, E. Josefsson, I. M. Jonsson, J. Hinds, H. H. Rees, J. A. Lindsay, A. Tarkowski, and M. J. Horsburgh. 2009. The Staphylococcus aureus response to unsaturated long chain free fatty acids: survival mechanisms and virulence implications. PLoS One **4**:e4344.
Pane-Farre, J., B. Jonas, K. Forstner, S. Engelmann, and M. Hecker. 2006. The sigmaB regulon in Staphylococcus aureus and its regulation. Int. J. Med. Microbiol. **296**:237-258.

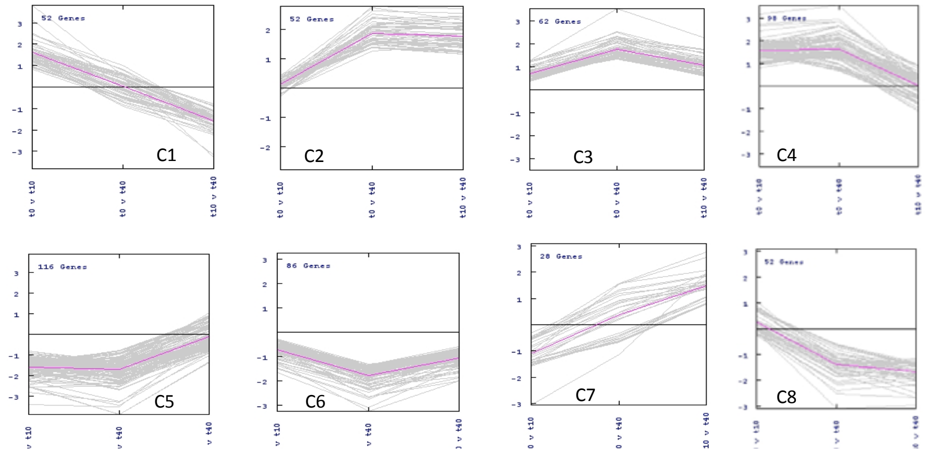

Supplement: Table S1 — Changes in global transcription of S. aureus COL in response to amicoumacin A. Hierarchical clustering has been done to classify the regulated genes based on their expression profiles. Genes in clusters 1–4 were upregulated and those in clusters 5–8 were downregulated. (PDF) [file pone.0034037.s001.pdf]
